# Impact of Bacterial Metabolites on Gut Barrier Function and Host Immunity: A Focus on Bacterial Metabolism and Its Relevance for Intestinal Inflammation

**DOI:** 10.3389/fimmu.2021.658354

**Published:** 2021-05-26

**Authors:** Naschla Gasaly, Paul de Vos, Marcela A. Hermoso

**Affiliations:** ^1^ Laboratory of Innate Immunity, Program of Immunology, Institute of Biomedical Sciences, Faculty of Medicine, Universidad de Chile, Santiago, Chile; ^2^ Immunoendocrinology, Division of Medical Biology, Department of Pathology and Medical Biology, University Medical Center Groningen, Groningen, Netherlands; ^3^ Advanced Center for Chronic Diseases (ACCDiS), Universidad de Chile, Santiago, Chile

**Keywords:** bacterial metabolites, gut immune barrier, inflammation, gut microbiota, dietary fiber, tryptophan, SCFAs, secondary bile acids

## Abstract

The diverse and dynamic microbial community of the human gastrointestinal tract plays a vital role in health, with gut microbiota supporting the development and function of the gut immune barrier. Crosstalk between microbiota-gut epithelium and the gut immune system determine the individual health status, and any crosstalk disturbance may lead to chronic intestinal conditions, such as inflammatory bowel diseases (IBD) and celiac disease. Microbiota-derived metabolites are crucial mediators of host-microbial interactions. Some beneficially affect host physiology such as short-chain fatty acids (SCFAs) and secondary bile acids. Also, tryptophan catabolites determine immune responses, such as through binding to the aryl hydrocarbon receptor (AhR). AhR is abundantly present at mucosal surfaces and when activated enhances intestinal epithelial barrier function as well as regulatory immune responses. Exogenous diet-derived indoles (tryptophan) are a major source of endogenous AhR ligand precursors and together with SCFAs and secondary bile acids regulate inflammation by lowering stress in epithelium and gut immunity, and in IBD, AhR expression is downregulated together with tryptophan metabolites. Here, we present an overview of host microbiota-epithelium- gut immunity crosstalk and review how microbial-derived metabolites contribute to host immune homeostasis. Also, we discuss the therapeutic potential of bacterial catabolites for IBD and celiac disease and how essential dietary components such as dietary fibers and bacterial tryptophan catabolites may contribute to intestinal and systemic homeostasis.

## Introduction

A wide range of inflammatory conditions has increased steeply in Western and developing countries, attributed to changes in the mucosal immune system, specifically the gastrointestinal tract. The mucosal immune system acts as a guardian, preventing invasions and preserving a healthy gut microbiota (GM). Additionally, GM is a determining factor in general health, releasing metabolites with anti-inflammatory properties that use intestinal homeostasis. Immune responses against microbial and dietary antigens can cause inflammatory disorders such as inflammatory bowel disease (IBD) and celiac disease (1).

Particularly, IBD is a recurrent, chronic and non-specific inflammatory bowel condition that includes ulcerative colitis (UC) and Crohn’s disease (CD), disorders characterized by intermittent periods of activity (mild, moderate, or severe) or inactivity (remission). IBDs are considered as an abnormal immune response and chronic intestinal inflammation caused by genetic and environmental factors, in addition to complex interactions between the GM and the host’s immune system, with development of specific treatments a challenging task (2). Alternatively, celiac disease is an autoimmune destruction of the villi of the small intestine triggered by gluten in genetically susceptible individuals, causing nutrient malabsorption, leading to intestinal and extra-intestinal symptoms, and a gluten-free diet (GFD) for life the only treatment. The pathogenesis is multifactorial, and although genetic predisposition occurs in 30% - 40% of the general population, only a small proportion of these individuals will develop the disease, suggesting the importance of environmental factors, with recent evidence supporting the participation of GM (3).

Therefore, this dynamic triad in the gut (mucosal immunity, GM, and diet) can be jointly explored, modulated, and enhanced for the prevention and treatment of many inflammatory diseases.

Here, we present an overview of current knowledge on host microbiota-epithelium-gut immunity crosstalk, focusing on how microbial-derived metabolites contribute to host immune homeostasis. Also, we discuss the therapeutic potential of bacterial catabolites for some inflammatory conditions, such as IBD and celiac disease and how essential dietary components, such as dietary fibers and bacterial tryptophan catabolites aid intestinal and systemic homeostasis.

## A Brief Description of Gut Microbiota and Host Immunity

GM corresponds to microorganisms (bacteria, archaea, fungi, viruses and phages) inhabiting the human gastrointestinal tract (4). The phylum Bacteroidetes and Firmicutes represent more than 90% of the GM bacterial component, including sub-dominant phyla such as Proteobacteria, Actinobacteria and Verrucomicrobia (5, 6). Complex GM interaction with the host immune system begins at birth, where microorganisms initiate immune development and the immune system subsequently orchestrates the GM composition (7, 8). This is a vulnerable period as the intestine is a critical site for multiple host-microbe interactions during life. Commensal microorganisms establish a relationship with the host, essential for immune system development and function through production of several types of metabolites. Next to producing metabolites the GM interacts with intestinal cells in the local environment which subsequently also impacts immune homeostasis (9).This interaction with epithelium also initiates production of numerous microbial metabolites (10) and stimulate signaling pathways, profoundly impacting gut health and also that of more distal organs (11).

Gut health is highly influenced by diet. Molecular structures in diet provide substrates for the host and GM, regulating GM composition and therewith gut-immune barrier function. However, how diet influence this also depends on other factors such as genetics and lifestyle (6). Via GM and diet-derived microbial metabolites diet can influence intestinal homeostasis (12), with emerging evidence showing diet and nutrients impact GM composition, production of microbial metabolites, and immune function; and that disturbances in nutrition may result in development of intestinal diseases such as IBD (13, 14).

The realization that GM and its metabolites have a strong influence on disease development also led to the realization that GM is an attractive target to prevent disease. The microbiome constitutes a collection of aggregated genomes and genes present in GM, modulating the host’s metabolism, influencing immune system performance, and thus altering our concepts of health, disease risk, and effect of western lifestyle (4). The host-GM interactions aim to establish immune tolerance while at the same time initiating strong defense responses toward dangerous microorganisms (15). The epithelium separates the intestinal lumen from underlying tissues, with a dense layer of mucus (16). This mucosal barrier organizes around hyperglycosylated mucin MUC2, protecting and limiting the immunogenicity of intestinal antigens, determining an anti-inflammatory state in mucosa embedded dendritic cells (17). Critical for transepithelial permeability are tight junctions (TJs) that restrict passage into the host of pathogens, microbes or toxins (18). GM metabolic signals, such as indole metabolites and short chain fatty acids (SCFAs) can strengthen the epithelial barrier by increasing expression of TJs and cytoskeleton-associated proteins (19). The importance of GM for epithelial integrity is illustrated in studies showing that GM alterations (dysbiosis) and intestinal barrier function disruption trigger a wide range of pathologies, including autoimmune, neurodegenerative and inflammatory disorders (13).

**Figure 1:**
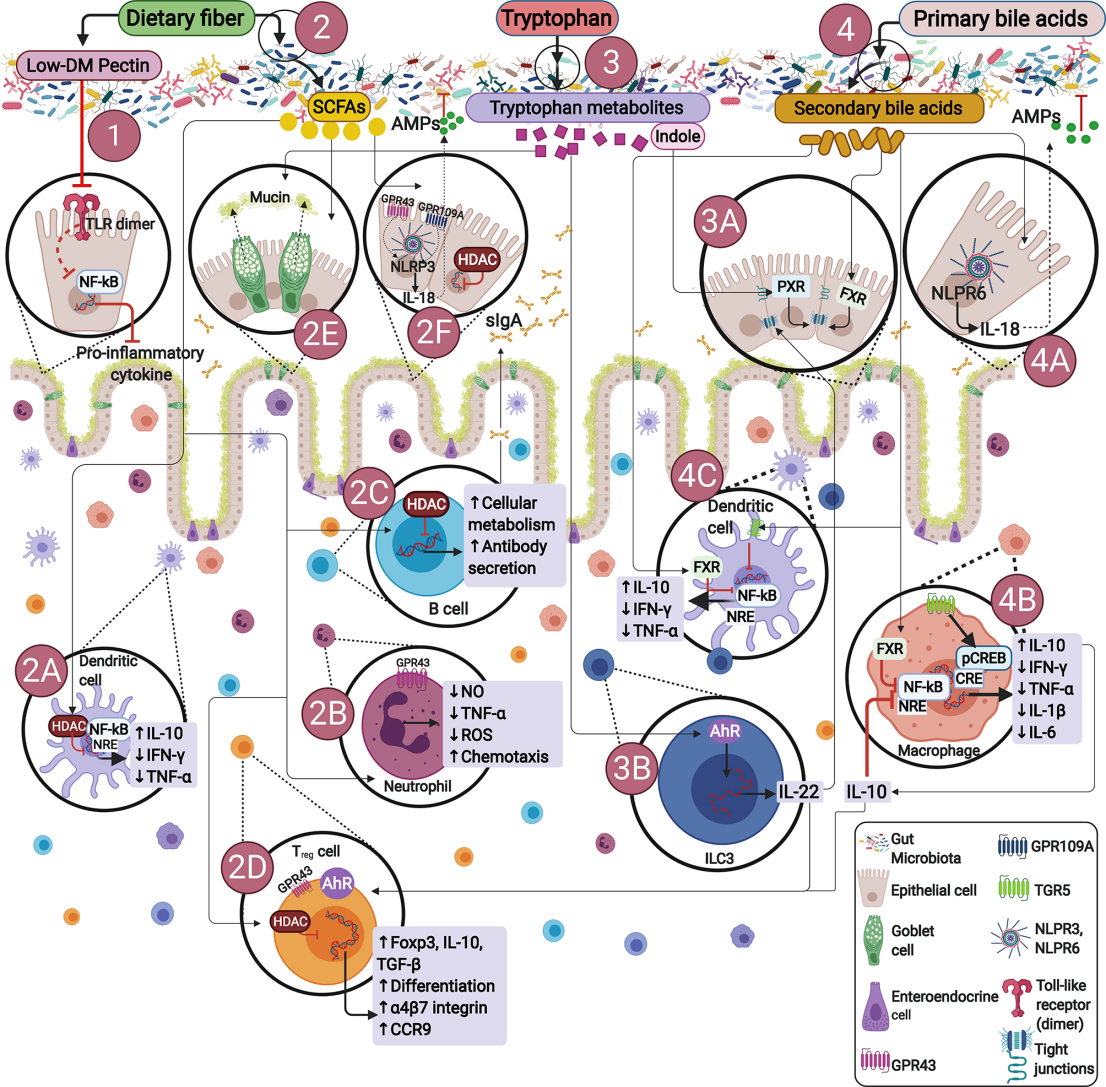
Endogenous or dietary compounds and dietary-derived bacterial metabolites effects on the gut immune barrier. (1) Direct effect of dietary fiber. Some dietary fibers have been shown to have direct effects on immune cells. E.g., low degree of methyl esterification (Low-DM) pectin binds to TLR2, inhibiting TLR2-1 heterodimer activation, thus reducing NF-κB activation. (2) Bacterial fermentation of dietary fiber by SCFAs-producing bacteria. SCFAs inhibit histone deacetylases (HDACs) and thus NF-kB-induced pro-inflammatory mediators: (2A) induce neutrophil chemotaxis by binding to GPR43, (2B) promote IgA secretion (2C), stimulate Tregs proliferation and differentiation by activating GPR43 and inhibiting HDACs (2D), influence NLRP3 by activating GPR43 or GPR109A facilitating IL-18 expression, thus promoting repair and maintaining barrier function (2E, 2F). (3) Bacterial tryptophan metabolism: Indole promotes epithelial barrier function through the pregnane X receptor (PXR). Bacterial tryptophan metabolites are AhR ligands in ILCs, ROR*γ*t interacts with AhR stimulating IL-22 expression (3A, 3B), IL-22 promotes antimicrobial peptide expression and enhances goblet cell proliferation for mucin secretion (2E). (4) Bacterial bile acid metabolites: Gut metabolites-derived from microbiota, endogenous or dietary compounds participate in microbiota-host interactions, exerting diverse effects on epithelial or immune cells through different signaling pathways. Secondary bile acids regulate epithelial integrity by binding to the farnesoid X receptor (FXR) in epithelial cells (4A), regulate macrophages differentiation into M2 profile *via* TGR5 and FXR activation, reversing inflammatory pathways producing IL-10 (4B), with further IL-10 production by Treg (2D). Additionally, TGR5 signaling involves NF-κB inhibition, and FXR signaling repressing NF-κB-responsive elements (NRE) in macrophages and dendritic cells (4B, 4C).

Additionally, the combination of genetic, environmental and dysbiosis factors favor the development of immune response alterations, promoting inflammatory diseases (20, 21). As described, immune signals generated by GM modulate commensal microorganisms (22, 23), protecting the host against opportunistic pathogens (23). Essentially, the host-GM interactions are mediated by certain endogenous, dietary compounds and dietary-derived bacterial metabolites, mainly stimulating B cells differentiation in order to produce immunoglobulin A (IgA), initiating formation of regulatory T cells (T_regs_), T helper 1 (T_h1_), T helper 17 (T_h17_), as well as group 3 innate lymphoid cells (ILC3s), among others, potentially preventing or developing inflammatory diseases (**Figure 1**).

## Direct or Indirect Mechanisms of Dietary Fibers and Bacterial Catabolites and Their Role in Health and Disease

### SCFAs and Health Effects

#### Dietary Fiber Catabolism

Dietary fiber (DF) are edible parts of plants or carbohydrates resisting digestion in the human small intestine, being partially or completely fermented by GM in the large intestine. The Codex Alimentarius defines DF as polymers of edible carbohydrates from natural sources such as cereals, fruits and vegetables, and those from raw materials by physical, enzymatic and chemical mechanisms or synthetic carbohydrate polymers with physiological benefits (24). Moreover, fermentation is influenced by many structural and physicochemical parameters. DF fermentation is associated with lower colonic pH, increased large intestinal bacterial mass, reduction of pathogens, stimulation of antioxidant compound and vitamin production, as well as regulation of epithelial barrier and immune system stimulation, among others.

One main DF classification refers to its solubility, with secondary physiological benefits on viscosity and fermentability, contributing to gel formation in the intestinal tract, and DF metabolized by GM, respectively (25). Insoluble DFs, such as lignin and cellulose, are poorly fermentable by GM, due to their inability to retain water. Soluble DFs, such as pectin, inulin, *β*-glucans, oligosaccharides, and guar gum are fermentable and constitute the main energy source for GM, and generally, are more fermentable with higher viscosity (26). Fermentation is possible in the presence of keystone species and strains possessing the enzymatic capacity to metabolize specific DF (24).

Interestingly, DF fermentation changes GM diversity, depending on the DF nature (27, 28), allowing production of SCFAs, amines, ammonium, gases, and phenols. Also it stimulates growth and diversity of GM and induces release of energy and water (29). A rich saturated fat and simple carbohydrate diet, but low in DF, is associated with increased risk for obesity, diabetes, cardiovascular disease, and colorectal cancer, conditions having low-grade systemic inflammation (30, 31). Concordantly, high DF intake correlates with reduced incidence of disease and mortality (32–35). DF preserves the gastrointestinal immune barrier (36, 37), with dysfunction triggering autoimmune disease and IBD development (13, 35, 38). These beneficial effects are attributed to fermentation products, mainly SCFAs, interacting with small intestinal immune cells before its degradation by microbial enzymes (39).

The GM and the mucosal immune system is a complex ecosystem, involving mutualistic GM-host relationship, where microorganisms convert diet carbohydrates, proteins, and fats into metabolites, having positive or negative health effects (40). The human digestive system lacks enzymes to digest complex polysaccharides, such as pectins, xylan or celluloses, consequently reaching the colon with their intact structure, and subsequently fermented by GM bacteria (41, 42).

GM has numerous enzymes, 16 families being carbohydrate esterases, 22 polysaccharide lyase, with 130 glycoside hydrolases standing out, allowing DF fermentation, giving GM flexibility to adapt to many substrates (24). Firmicutes and Actinobacteria phyla are the main bacteria responding to DF, although few enzymes initiate substrate degradation (43). The main substrates for bacterial fermentation and SCFA production are inulin, wheat and oat bran, cellulose, guar gum, pectin, and resistant starch; the latter an important source for butyrate production (2). Phylum Bacteroidetes members are major acetate and propionate producers, while phylum Firmicutes produce mostly butyrate (44, 45).

The main human intestinal butyrate-producing bacteria are *Faecalibacterium prausnitzii* and *Clostridium leptum*, Ruminococcaceae family members, and *Eubacterium rectale* and *Roseburia* spp from the Lachnospiraceae family (44, 45). Additionally, sugar and/or lactate utilizing bacteria such as *Eubacterium hallii* and *Anaerostipes* spp. produce butyrate from lactate and acetate in a cross-feeding process (44). However, members of the phyla Fusobacteria, Proteobacteria, Actinobacteria, Spirochaetes and Thermotogae are potential butyrate producers, expressing genes encoding butyrate synthesis enzymes, such as butyryl-CoA dehydrogenase, butyryl-CoA transferase and/or butyrate kinase (46).

Production of other SCFAs is determined by Actinobacteria, such as the *Bifidobacterium* species, producing lactate during DF fermentation (47). Bifidobacteria metabolizes low molecular weight carbohydrates; *Bifidobacterium longum* subsp. *infantis* prefers short-chain fructo-oligosaccharides (48), additionally, a relevant phylum Verrucomicrobia member, the mucin degrader, *Akkermansia muciniphila*, produces acetate and propionate (45, 49).

Although metabolite production depends on the bacterial species capable of fermenting DF, their consumption is a good way to stimulate metabolite production through modulation of the GM composition.

#### SCFAs and Receptors

SCFAs are carboxylic acids produced by DF fermentation, having 1-6 carbon aliphatic tails, with acetate (C2), propionate (C3) and butyrate (C4) being the most abundant (2). Soluble DF rich diets are associated with less inflammation due to higher SCFAs production and stimulation with G-protein-coupled receptors (GPCR), i.e., the primary receptor for SCFA. GPCR inhibits NF-kB activation in immune cells and intestinal epithelial cells (IEC) (50). SCFAs profoundly impacts human health by being an energy for colonocytes, regulating glucose metabolism, fatty acids and cholesterol hepatic biosynthesis, inhibiting pathogen growth and reducing intestinal inflammation (51) and enhancing barrier function both *in vitro* and *ex vivo* (52–54) by altering TJs formation (**Figure 1**) (53, 55). An colonocyte energy source, butyrate is the most relevant SCFAs bacterial metabolite, regulating gene expression and inflammatory (40). Studies evaluating butyrate effect on intestinal permeability, both *in vitro* and *ex vivo*, indicate low concentrations of butyrate are beneficial, while excessive luminal concentration could cause mucosal barrier disruption (52, 53). It has been demonstrated that high concentrations of SCFAs induces intestinal mucosa injury in newborn rats, but although maturation dependent, this injury disappears in mature mucosal defense (56). Additional studies (52) show that high butyrate concentrations disrupt assembly of TJs to the intestinal barrier, proposing that barrier function regulation by low butyrate concentrations in Caco-2 cell monolayers facilitates TJs assembly in a dynamic process mediated by AMP-activated protein kinase (AMPK) activation (53).

Both DF and butyrate are important components and metabolites involved in managing inflammatory diseases (57). SCFAs suppress IL-8 secretion and expression (58–61), although, high butyrate doses has the opposite effect (61). Understanding butyrate action in reducing inflammation is important, especially the histone acetylation mechanism (62), requiring more study as butyrate metabolizes to Acetyl-CoA, activating histone acetyltransferase (HAT), affecting cell turnover, structure and function and tumoricity, although with paradoxical effects (63, 64). Butyrate permeate the cell membrane by passive diffusion or is absorbed by apically proton-coupled monocarboxylate transporter 1 (MCT1) and sodium-coupled monocarboxylate transporter 1 (SMCT1) on IECs and/or immune cells (65). MCT1 primarily transports butyrate and is induced by SCFAs, inhibiting HDACs through MCT1 (66).

Cellularly, SCFAs activate signaling pathways through GPCRs: GPR41, GPR43 and GPR109A (expressed in colonic epithelium enteroendocrine cells, polymorphonuclear immune cells and smooth muscle cells) with different affinities **(** **Figure 1** **)**. GPR41 and GPR43 have a role in the immune surveillance of the colonic mucosa towards microbial activity (67), whilst GPR109A inhibits NF-kB activation as a tumor suppressor (68). Active absorption of butyrate is reduced in IBD with decreased MCT1 expression in inflamed mucosa of UC patients (69), possibly due to high TNF-α levels (70) or reduced butyrate-producing bacteria, causing faulty oxidization (71, 72), potentially indicating that butyrate reduces IL-8 expression mediated by GPR109A.

GPR41 and GPR43 activate the NLRP3 inflammasome (73), triggering IL-1*β* and IL-18 secretion (74, 75), and GPR43 regulates immune cells in experimental inflammation (36, 37), with sodium butyrate increasing disease severity in DSS model of germ-free mice (76). Butyrate modulates immune cells, such as macrophages (77), dendritic cells (78) and lymphocytes (77), and inhibits cytokines, such as IL-12p70 and IL-23, polarizing naïve CD4^+^ T cells towards the Th1 and Th17 subtypes (79). Butyrate affects the mucosal immune system through Treg expansion, and inhibits HDAC activity in dendritic cells, resulting in retinaldehyde dehydrogenase 1 (RALDH1) and GPR109A expression (77). RALDH1 modulates naïve T cell differentiation towards Treg producing IL-10 (77, 80, 81), with butyrate also enhancing Foxp3^+^ expression and Treg differentiation, reducing inflammation (80).

GM in IBD patients is characterized by loss of intraindividual diversity, with higher abundance of Proteobacteria and lower of Firmicutes. Interestingly, butyrate-producing bacteria *Faecalibacterium prausnitzii*, *Ruminococcus torques*, *Roseburia inulinivorans*, *Blautia faecis*, and *Clostridium lavalense* are reduced in patients (82–85), with lower luminal butyrate concentrations (86) and higher C-reactive protein, reflecting increased inflammation (84). Furthermore, a reduced butyrate-synthesis capacity has been described in IBD patients, with butyryl-CoA:acetate CoA-transferase (BCoAT) gene content inversely associated with disease activity and dysbiosis, being more evident in CD, and may relate to reduced DF intake (87). Alternatively, celiac disease associates with GM alterations, although no SCFA quantity differences or composition exist between celiac patients and healthy patients (88). Interestingly, prolonged administration of oligofructose-enriched inulin in children with celiac disease (under a gluten-free diet), has a moderate effect on GM composition, while SCFA production is stimulated in celiac children (89).

Accordingly, activated NF-kB in UC patients colonic mucosa is reduced by butyrate and SCFAs enemas (100 nM), directly correlating with disease activity (90, 91), with no effect in patients in remission (92). Depending on butyrate physiological concentration, it can be beneficial, with fewer molecules reaching colonocytes, as mucus layer prevents excessive concentrations towards epithelial cells, and increasing MUC2 expression (93). Regarding the above, the fact that butyrate effect is variable, is related to the physiological irrigation of this SCFA. At these or other doses, it is likely that in an *in vivo* IBD model, fewer butyrate molecules reach the colonocytes than in an *in vitro* model, since the colonic epithelium is covered by a thick mucus layer preventing passage of excessive concentrations of butyrate into the cell. Consequently, as butyrate concentration gradients run from the crypt-lumen axis and the luminal axis proximal-distal, colonocytes at the crypt base are exposed to an estimated µM concentration range, thus explaining its variable effects on inflammation.

#### Dietary Fiber and TLRs Interaction: Direct Effects

Certain DF have a direct anti-inflammatory effect by directly interacting with pattern recognition receptors (PRRs), mainly Toll-like receptor (TLR) 2 and 4, or Galectin-3 in intestinal immune barrier cells. Accordingly, low-grade methyl esterification pectins (Low-DM) block innate immune receptors, with pectin directly inhibiting pro-inflammatory TLR2-TLR1 pathway (without affecting TLR2-TLR6 tolerogenic pathway *in vitro*) (**Figure 1**) and preventing clinical symptoms in a TLR-2 dependent doxorubicin-induced ileitis murine model (65). Additionally, low-DM citric pectin suppresses TLR-induced inflammatory cytokine expression in a mouse model of endotoxin shock (94). Moreover, apple pectin oligo-galactan exhibits a protective efficacy in a murine model of dextran sulfate sodium (DSS)-induced colitis, decreasing LPS-induced TNF-α, and is probably associated to a mechanism comprising TLR4 internalization and redistribution (95). Similarly, resistant starch (RS) also binds predominantly to TLR2 and/or TLR5 in dendritic cells, affecting PRR signaling (96).

Pectin directly interacts with TLR2, inhibiting the proinflammatory signaling pathway, thereby contributing to barrier function and preventing colorectal cancer development (97). Similarly, *β*2 fructans also regulate mucosal homeostasis through activation of TLR2 (to a lesser degree TLR4, TLR5, TLR7, TLR8 and Nucleotide Binding Oligomerization Domain Containing 2; NOD2) producing IL-10/IL-12 in mononuclear cells (in chain length-dependent manner) (98), and regulating barrier function in T84 cells (99). Wheat bran and peanut fiber also improve intestinal barrier function, changing GM composition and TLR2 expression in pigs (100). Likewise, chicory pectin and intact guar gum DF exhibit an anti-inflammatory effect in a murine model of DSS-induced colitis decreasing proinflammatory expression (101, 102). Additionally, bengkoang DF binds to TLR4, favoring phagocytic activity in J774.1 macrophages (103).

Soluble DF has immunomodulatory effects by influencing human peripheral blood mononuclear cells (PBMCs) functions (independent of SCFA production) (39), and has symbiotic effects with certain *Lactobacillus*, synergistically stimulating IL-6 and IL-8 production in immune cells (104). Inulin and short-chain fructo-oligosaccharides (scFOS) directly promote, in a GM-independent manner, specific intestinal barrier function in a damage-induced model of Enterohemorrhagic *E. coli* (105).

All these fibers can be instrumental in prevention or lowering symptoms of chronic inflammatory conditions where the aforementioned gut epithelial processes are involved. Examples are celiac disease which results from oral tolerance to gluten breakdown in subjects carrying HLA-DQ2 or HLA-DQ8 variants (106). High maternal fiber intake during pregnancy is associated with a lower risk for celiac disease in children, while gluten intake associates with a high risk, suggesting that high dietary fiber and low gluten intake during pregnancy is a protective factor for celiac disease (107). The mechanisms are not yet clear but might be associated to the aforementioned protective effects of DF on gut epithelial immune barrier function.

Galectin-3, extracellularly or intracellularly expressed in immune and epithelial cells, plays a fundamental role in health and disease (108), interacting with carbohydrates and intracellular proteins. Galectin-3, acts as a PRR inducing innate responses against pathogens (109), enhanced by pectins (109), through galactose residues or arabinose side chains with rhamnogalacturonan I (RG-I) and rhamnogalacturonan II (RG- II) (110).

### Secondary Bile Salts and Health Effects

#### Secondary Bile Salts Catabolism

Primary bile acids (BAs) synthesized in the liver from cholesterol, conjugate to glycine or taurine before released and concentrated into bile in the gallbladder, and eventually secretion (111). Approximately 95% of BAs are reabsorbed in the ileum and recirculate to the liver, while a smaller percentage reach the colon, and are metabolized by GM or excreted; additionally, GM regulates BAs synthesis and uptake (112). In healthy people, the amount of daily secreted BAs depends on eating habits, with levels fluctuating between 200-600 mg per day (113) BAs metabolism by GM, and its effects on health and disease recently begun to be considered (114, 115), with two BAs being synthesized by the liver, colic and chenodeoxycholic acid. GM-mediated BAs transformations include deconjugation *via* bile salt hydrolases (BSH) hydrolyzing the amide bond and transforming deconjugated primary BAs to secondary BAs, mainly by 7*α*-dehydroxylation reactions exerted by a limited number of bacteria (116).

While deconjugation is performed by numerous bacteria, BSH are encoded in different genus species: *Lactobacillus, Bifidobacterium, Bacteroides* and *Clostridium*, being more diverse in Firmicutes (117). Conversion of primary to secondary BAs by 7*α*-hydroxylation is a relevant microbial BAs transformations in humans (118), as cholic acid is transformed to deoxycholic acid (DCA) and chenodeoxycholic acid to lithocholic acid (LCA). 7*α*-dehydroxylation, is a characteristic of *Clostridium* (*Clostridium hylemonae* and *C. scindens*, the latter important in the production of LCA), as well as *Eubacterium* (119, 120). Bacterial BAs metabolism plays a fundamental role in systemic and intestinal health, since imbalances in BA-GM crosstalk associates with inflammatory and gastrointestinal disorders (121).

#### Secondary Bile Salts and Receptors

Secondary BAs are potent nuclear receptor ligands, binding to farnesoid X receptor (FXR), vitamin D receptor (VDR) and pregnane X receptor (PXR), additionally acting as endogenous agonists for the microbial G protein-coupled bile acid receptor (TGR5). These receptors play an important role in BAs synthesis, regulation and metabolism (122) and are relevant in different pathological processes (**Figure 1**). BAs receptors are expressed in IECs, hepatocytes, liver parenchyma, muscles, neurons, among others (123), as well as in immune cells (124–127), responding to endogenous and bacterial antigens. In BAs metabolism by FXR, biliary acidosis inhibition by CYP7A1 inhibition is an important step, occurring *via* the hepatic bile salt export pump, inducing small heterodimer partner (SHP) expression. SHP inactivates the hepatic homologous receptor-1 (LRH-1) inhibiting CYP7A1 expression (128). Alternatively, FXR activated by BAs induces FGD15/19 to FGFR4 binding, inhibiting CYP7A1 (129).

During inflammation, FXR regulate intestinal immune responses driven by GM, with low-grade chronic inflammatory disorders associated to dysbiosis and altered BAs profiles in humans (130). IBD patients exhibit a low content of bile salt biotransformation genes (mostly from Firmicutes phylum) as well as low secondary and high primary BAs levels compared to healthy subjects (131, 132). Also FXR null mice are unable to reduce intestinal inflammation, promoted by GM-LPS stimulation of NF-kB and inflammatory cell recruitment (133). Additionally, antibiotic-induced GM alterations allow *C. difficile* spore germination and inhibit BAs-producing bacteria, depleting BAs production (134) and, thus inhibit FXR activation. Additionally, FXR deficiency in a Apc^Min/+^ and azoxymethane (AOM)-DSS murine model of colon carcinogenesis (associated with chronic colitis), resulted in premature death and increased tumor progression through Wnt signaling pathway activation in macrophages, neutrophils (135). Moreover, BAs deficiency (due to bile duct obstruction) associates with bacterial overgrowth and translocation, intestinal damage, reverted by BAs or GW4064 (FXR agonist) administration (136). Obeticholic acid (OCA) also a FXR agonist, decreases severity, maintaining epithelial barrier integrity and decreasing inflammatory cytokine production in a DSS or 2,4,6-Trinitrobenzene sulfonic acid (TNBS)-induced colitis murine model (125, 137), whilst isoDCA (secondary BA) induces peripheral Treg cells (138). Therefore, FXR rationally becomes a therapeutic IBD target, although, clinical trials using FXR agonists will be required.

GM activates TGR5 expression, affecting enteroendocrine cells, exhibiting immunomodulatory and anti-inflammatory effects (139), mainly in macrophages and monocytes (139). Importantly, TGR5 regulates energy metabolism and glucose homeostasis: inducing GLP-1 secretion, glucose metabolism, intracellular cAMP levels, transcription of type 2 iodothyronine deiodinase (Dio2) gene (encoding type 2 deiodinase (D2) converting thyroid hormone T4 into triiodothyronine, T3). Through TGR5 activation, BAs inhibit production of pro-inflammatory mediators IL-1, IL-6 and TNF-*α*, induced by LPS (140). Likewise, BSH-containing bacteria alters TGR5-mediated pro-inflammatory or anti-inflammatory activity, dissociating taurine or glycine from BAs.

TGR5 agonist effect associates with intestinal IL-10 increase (141). BAR501, a selective agonist of TGR5, protected mice from colitis by polarizing intestinal macrophages from M1 to M2 phenotype and reducing intestinal and circulating monocytes/macrophages (141, 142).

In celiac disease, immunologically mediated extraintestinal manifestations in the liver can occur. Both gut and liver, share lymphocyte recruitment and return routes, with gut T lymphocytes contributing to liver and biliary inflammation (143). The gut-liver axis describes a close metabolic and immunological connection between the intestine and the liver, and an imbalance or damage in this axis can trigger innate immune responses that induce liver damage or contribute to its progression (144). It should be noted that in celiac disease, the intestinal permeability, together with chemokine CXCR3, increase entry of food and bacterial antigens, as well as bacterial metabolites *via* the portal vein, triggering immune responses by activation of PPRs (CD14/TLR4 complex, inflammasome, etc.), generating liver inflammation and metabolism alterations. Alternatively, dysbiosis in this pathology produces an increase in intestinal permeability and mediates inflammatory processes, enhancing celiac disease immunopathology and altering BAs composition, impacting on hepatic FXR and TGR5, inflammation and BA metabolism (145, 146).

The above demonstrates a clear role of GM-BAs-FXR/TGR5 axis in Tregs, monocytes and macrophages regulation, highlighting the potential use of secondary BAs or other natural or synthetic ligands as new therapies in inflammatory pathologies.

## Tryptophan Metabolism and Health Effect

### Tryptophan Catabolism

Dietary proteins and peptides are normally digested in the small intestine, resulting in free amino acids actively absorbed from the intestinal lumen into the systemic circulation. Next to serving as nutritional source, proteins can serve as GM fermentable substrates when escaping from digestion in the small intestine and reaching the large intestine. Tryptophan (Trp) is an essential amino acid composed of a *β*-carbon, connected at position 3 of an indole group, naturally provided by poultry, milk, tuna, fish, cheese, bread, oats, prunes, chocolate, and peanuts. Of the 20 amino acids, Trp has the highest molecular weight and is a biosynthetic precursor of a large number of metabolites (147). Alternatively, it can be metabolized by the host in the kyneurine pathways or be utilized for serotine synthesis in the brain and intestine; or by the GM, which can directly use Trp, partially limiting its availability, metabolizing approximately 4-6% indole, skatole, tryptamine and indole acid derivatives (148). Intestinal bacterial species convert Trp to tryptamine and indole-3-pyruvic acid (IPyA), additionally converting it to indole, indole-3-acetaldehyde (IAAId), and indole-lactic-acid (ILA). Furthermore, IAAId converts to indole-3-acetic acid (IAA) and tryptophol, and IAA indirectly converts to skatole through decarboxylation (148, 149). Lastly, ILA can convert to indole-acrylic acid (IA) and subsequently to indole propionic acid (IPA) (150). Different bacterial species possess different catalytic enzymes, some cooperating with each other to generate a Trp metabolite (**Table 1**); for example, indole is produced by Firmicutes phylum members such as *Enterobacter aerogenes*, *Clostridium limosum*, *C. tetani*, *C. lentoputrescens*, *C. bifermentans*, *C. melanomenatum*, as well as some members of the Bacteroidetes, Fusobacteria and Proteobacteria phylums; alternatively, IAID is produced in a reduced number of species belonging to Firmicutes phyla, such as *Lactobacillus johnsonii*, *L. reuteri*, *L. murinus* and *L. acidophillus* (**Table 1**). The Trp bacterial metabolism is a complex process, with many bacterial strains having catalytic capacity against Trp, but many of them remain to be identified. Accordingly, understanding which bacterial consortia produce Trp metabolites is necessary, and considering the GM intervariability (151) will allow the design of targeted strategies to steer Trp metabolite production modulating GM composition through the diet.

**Table 1:** Gut bacterial species and enzymes involved in bacterial Tryptophan metabolism.

Trp Metabolite	Phylum	Bacterial species	Enzymes	Model	Refs.
Indole	Firmicutes	*Enterobacter aerogenes, Clostridium limosum, C. tetani, C. lentoputrescens, C. bifermentans, C. melanomenatum*	Tryptophanase	Human gut content (150) Bacterial culture (152)	(150, 152–154)
	Bacteroidetes	*Bacteroides ovatus, B. thetaiotaomicron*			
	Fusobacteria	*Fusobacterium nucleatum*			
	Proteobacteria	*Escherichia coli, E. albertii*			
Indole-3-acetic acid (IAA)	Firmicutes	*Lactobacillus reuteri, C. difficile, C. paraputrificum, C. lituseburense, C. paraputrificum, E. cloacae, Peptostreptococcus asscharolyticus*	Indolepyrubate decarboxylase	Human gut content (150) Bacterial culture (155)	(150, 155)
	Bacteroides	*Parabacteroides distasonis, B. fragilis. B thetaiotaomicron, B. eggerthii, B ovatus.*			
	Proteobacteria	*E. coli*			
	Actinobacteria	*Bifidobacterium pseudolongum, B. adolescentis, B. longum subsp. Longum*,			
Indole propionic acid (IPA)	Firmicutes	*C. botulinum, C. caloritolerans, C. paraputrificum, C. sporogenes. C. cadaveris, Rauschbrand bacillus.*	Pyruvate:ferredoxin oxidoreductase	Human stool samples, DSS-induced colitis mice, cell culture (156)	(154–156)
	Bacteroidetes	*P. anaerobius, P. russelli, P. stomatis*	Phenyllactate dehidratase	Human gut content (150) Bacterial culture (152, 157)	(150, 152, 157)
Indole-acrylic acid (IA)	Bacteroidetes	*P. anaerobius, P. russelli*	Phenyllactate dehydratase	Human gut content (150) Bacterial culture (152, 157)	(150, 152, 157)
Indole-3-aldehyde (IAID)	Firmicutes	*L. johnsonii, L. reuteri, L. murinus, L. acidophillus.*	Aminotransferase	Murine model (158, 159)	(158–160)
Indole-lactic-acid (ILA)	Firmicutes	*C. saccharolyticum, Eubacterium cylindroides, P. asaccharolyticus.*		Human gut content (150) Bacterial culture (155, 161)	(150, 155, 161)
	Bacteroidetes	*B. ovatus, B. thetaiotaomicron*			
	Proteobacteria	*E. coli*			
	Actinobacteria	*B. pseudolongum, B. adolescentis*			
Indole-3-acetamide (IAM)	Proteobacteria	*Burkholderia pyrrocinia*	Tryptophan-2-monooxygenase	Bacterial culture (162)	(162)
3-Metyl-indole (skatole)	Bacteroidetes	*B. thetaiotaomicron*		Human gut content (150) Bacterial culture (152, 157)	(150, 152, 155)
	Firmicutes	*E. rectale, Butyrivibrio fibrisolvens*			
Tryptamine	Firmicutes	*Ruminococcus gnavus, C. sporogenes*	Tryptophan decarboxylase	Bacterial culture (152, 163)	(152, 163)
Indole-3-pyruvic acid (IPyA)	Firmicutes	*C. bartlettii, C. sporogenes*	Tryptophan aminotransferase	Bacterial culture (152)	(152)

As will be outlined in next sections of this review, Trp metabolites have many beneficial effects for gut epithelial barrier function.

A main Trp metabolite is indole (conserved molecule between kingdoms and species). It is produced by bacteria for biofilm formation, regulating bacterial motility and resistance against non-indole producing species such as *Pseudomonas aeruginosa jhonsonii* and *Salmonella enterica* (164). Using the enzyme tryptophanase, bacteria converts Trp to indole and numerous indole-derived metabolites such as IAID, IAA, ILA, and IA (**Table 1**) (165).

The endogenous and microbial Trp pathway (the kyneurine pathway), and the indole microbial pathway converge on the xenobiotic receptor, AhR, (located in the cytosol of resting cells) and when bound to its ligand, translocates into the nucleus heterodimerizing with AhR nuclear translocator (ARNT), binding to dioxin-responding elements within enhancer and target gene promoter.

Bacterial Trp metabolites are low-affinity ligands for AhR, the most effective being indole, skatole, tryptamine, IPyA, IA and Indole-3-acetamide (IAM); whilst IAA, IPA, ILA and IAID the least active (166). Additionally, methyl-indoles and methoxy-indoles have synergistic effects *in vitro* (167), with different bacterial metabolites, such as SCFAs and secondary BAs.

Trp is an important regulator of inflammatory responses in mammals (**Figure 1**) (168), such as mice and pigs, with a low Trp diet making mice more susceptible to chemically induced inflammation (169). Also, it regulates bone remodeling, carcinogenesis, organ development, neurophysiology, metabolic diseases and, xenoprotection (170–174). As a mucosal protection against inflammation, *Lactobacillus reuteri* uses Trp to expand and generate an AhR ligand: IAID, contributing to IL-22 transcription in innate lymphoid cells and T cells, allowing a balanced GM.

### Indoles and Receptors

Bacterial indoles, such as IPyA, increase intestinal barrier function through PXR, decreasing TNF-α expression in enterocytes, whilst promoting goblet cell differentiation in female C57BL/6J mice (156). PXR^-^/^-^ mice show increased intestinal permeability and enhanced TLR4 expression, demonstrating direct chemical communication between gastrointestinal symbionts and PXR pathway in mucosal homeostasis (174). Interestingly, IPyA reduces intestinal permeability in high-fat diet mice, indicating that obese subjects with low IPyA levels have an imbalanced intestinal barrier function (175). Additional to intestinal barrier function and inflammatory response regulation, IPyA promotes goblet cell differentiation in mice (156).

Absence of Trp metabolizing bacteria impact on IL-22 immune regulation, with reduced IL-22 production seen in IBD. Additionally, GWAS studies in IBD patients have identified mutations impacting IL-22 pathways, especially in the Card9 gene sensing type-C lectins, as also witnessed in Card9 deficient mice, prone to DSS-induced colitis (176, 177). Furthermore, IBD patient´s carrying CARD9 polymorphism have reduced faecal AhR activity, Trp levels and GM production of AhR ligands, making GM an attractive target for modulation (176, 177). Concurrently, reduced serum Trp levels in patients negatively correlates with disease activity or C-reactive protein levels (178), although, determining if GM modification or indole-producing bacterial populations affect IBD severity is unknown.

AhR defective signaling contributes to pathogenic responses, witnessed in celiac disease patients, with AhR transcript decreased in IECs and peripheral mononuclear cells, although, an AhR agonist (6-Formylindolo(3,2-b)carbazole; Ficz), reduced pro-inflammatory cytokine, granzyme B and perforin expression *in vitro*, and reverted intestinal injury in a poly I:C-induced-celiac disease murine model (179). AhR ligands production by GM decreases gluten immunopathology in non-obese diabetic (NOD) mice expressing DQ8 (a susceptible gene to celiac disease; NOD/DQ8), as seen with high-Trp diet, *L. reuteri* or Ficz administration. Reduced AhR ligands production by fecal microbiota and low AhR activation in active celiac disease patients, highlights the role of GM modulating AhR pathway, becoming a new therapeutic strategy for treatment (180).

## Bacterial Catabolites as Aryl Hydrocarbon Receptor (AhR) Ligands: Key Role in Gut Microbiota and Immunity

AhR is a ligand-dependent transcriptional factor widely expressed in immune, epithelial, endothelial, and stromal cells in barrier tissues, and extensively studied in response to chemical contaminants. AhR senses a wide range of intestine signals, maintaining homeostasis between GM and host (181–183), activating proliferation of colonic stem cells, epithelial barrier functions, and regulating immune cells, such as ROR*γ*t^+^ intraepithelial innate lymphoid cells (ILC3), T helper lymphocytes (Th 17/22), *γ* *δ*T lymphocytes, antigen presenting cells and Foxp3^+^ Treg lymphocytes (151, 184–187). AhR response to physiological ligands has become an interesting focus of research, and the next section reviews current evidence on its molecular interaction and function in the immune system in steady state and intestinal inflammation, and its relationship with GM.

In addition, independent of GPCRs or MCT1 pathways, butyrate activates the AhR, modulating the interaction between diet, GM and host (151, 184, 185) therefore is reduced in IBD Thus, butyrate, by inhibiting HDACs, increases CYP1A1 expression in a AhR-dependent manner in Caco-2 and HT-29 cells (188).

### Role of AhR in Intestinal Barrier Function and Intestinal Immune Cells

Canonical (181), and non-canonical AhR signaling pathway have been described, either at the genomic or non-genomic level through association with other transcription factors, such as NF-kB, or Src kinase. Inactive AhR joins to Src kinase in the cytosol, when an agonist interacts with AhR, a conformational change occurs allowing (170, 189–191). Non-genomic AhR signaling is responsible for at least two anti-inflammatory outcomes, LPS exposure induced suppressive effects mediated by AhR-associated Src kinase phosphorylation of indolamine 2,3-dioxygenase IDO1, inducing TGF-β transcription in dendritic cells (151), or IL-10 production (192). Interestingly, after AhR activation by Ficz exposure, CD8αα^+^ TCRαβ^+^ intraepithelial lymphocytes (IELs) resist apoptosis with AhR, IL-15 and IL-10 up-regulation, and IFN-γ reduction in a colitis model (193). In IBD, immune cells express low levels and altered activity of AhR, maintained by decreased GM-derived AhR ligand production, mainly bacterial Trp metabolites and butyrate (151). Accordingly, depleted dietary AhR ligands alter GM composition and function (194), with Trp metabolites present in murine cecal content and human feces, activating AhR, and are considered intestinal activity biomarkers (182).

A novel AhR activator is 1,4-dihydroxy-2-naphthoic acid (DHNA), a precursor of vitamin B12 produced by *Propionibacterium freudenreichii* ET-3, inducing intestinal anti-microbial protein synthesis, altering GM composition and inhibiting murine DSS-induced colitis (196). Similarly, indole-3-ethanol, indole-3-pyruvate and indole-3-ethanol protect against increased gut permeability in a murine DSS-induced colitis model (195). AhR maintain intestinal permeability, with Ficz promoting goblet cell differentiation through AhR-pErk1/2 signaling pathway, and ameliorating DSS-induced colitis (197). Furthermore, after intestinal reperfusion ischemia and hypoxia, AhR activation increases Notch1 signaling, thus reducing epithelial barrier dysfunction *in vivo* and *in vitro* (198). The epithelial barrier function is regulated by direct (AhR activation by Ficz inhibited Par-6 expression through the Ap-2*γ* pathway) (199) and indirect mechanisms (AhR activation by Ficz prevented TNF-α/IFN-*γ*-induced decrease in TJ disruption) (200), with AhRs increasing TJ protein expression in response to IL-22 produced by CD4^+^ T cells (201). Finally, AhR expression in IELs is abrogated by miR-124 in active CD patients (202), therefore, AhR inhibits pro-inflammatory pathways in IECs preserving intestinal permeability.

### Macrophages as Potential Target in Intestinal Inflammatory Disorders

Intestinal macrophages are central in establishing and maintaining mucosal homeostasis, with its dysregulation generating loss of tolerance towards dietary antigens and GM, underlying IBD chronic inflammation. Together with SCFAs, bacterial Trp metabolites control susceptibility to intestinal inflammation through AhR activation pathways in macrophages. AhR deletion in intestinal mucosa CD11c^+^dendritic cells and certain macrophage subsets increases the stem cells in the ileal epithelium and differentiates epithelial precursors, causing greater susceptibility to murine DSS-induced colitis (203). Furthermore, a recent study observed that I3C treatment in C57BL/6 mice reduced macrophages expressing F4/80, demonstrating a diminished infiltration of innate immune cells into the colon. This effect was not observed in Ahr-/- mice, suggesting that the therapeutic effects of I3C was AhR-dependent (204). Alternatively, a synthetic pelargonidin (water soluble anthocyanidin) can be transactivated by the AhR, attenuating pro-inflammatory activities of Raw264.7 macrophages in an AhR-dependent manner. The administration of this compound resulted in a dose-dependent attenuation of TNBS-induced colitis in Balb/c mice and promoted M2 macrophage expansion (205).

AhR negatively regulates LPS-mediated inflammatory responses in macrophages, inducing IL-6 and TNF-α production in AhR-deficient macrophages compared to WT cells. Furthermore, AhR^-^/^-^ mice are more sensitive to LPS-induced lethal shock, similarly seen in STAT1 deficient animals, indicating that AhR complexes with STAT1 and NFκB in LPS-stimulated macrophages (206).

Accumulation of anti-inflammatory profile (M2) macrophages in the intestinal microenvironment appears to be crucial in restoring tissue homeostasis (207). Diet-derived bacterial ligands capable of modulating macrophages towards an M2 phenotype, AhR agonist-producing probiotics, or synthetic AhR agonist analogs (designed to optimize their anti-inflammatory activities) administration, could provide future anti-inflammatory therapeutic approaches.

## Future Directions

GM profoundly influences the inflammatory state *via* production of immunomodulating metabolites, making it an attractive target for therapeutic manipulation, using well-founded microbe- and/or metabolite-based therapies. Evidence discussed in the previous sections proposes various challenges and future directions to follow, for example:

- DF exhibit diverse effects on epithelial integrity, macrophages and dendritic cells (DCs) responses and is instrumental in enhancing the intestinal immune barrier through stimulating GM interaction with epithelium. However, to better understand DF impact, human studies controlling factors, such as diet, DF functional or nutraceutical-based foods to limit disease development are needed.
- Both IBD and celiac disease are characterized by alterations in the GM composition, SCFAs production, energy supply to colonocytes and local mucosal inflammation. Thus, GM empirical modulation can increase SCFAs-producing bacteria *in vitro* and *in vivo*, enriching its diversity, demonstrating clinical and histological improvement.
- Modulation of GM and secondary BAs profile represent novel therapeutic approaches to manage intestinal diseases such as IBD and colorectal cancer. Future research could elucidate secondary BAs effects on their receptors revealing opportunities for prevention and control of inflammatory diseases.
- The connections between Trp catabolites and human health are promising but needs further studies. By understanding their dynamics and functional implications for stimulating anti-inflammatory pathways mediated by PXR and AhR, maintaining intestinal barrier integrity, novel opportunities for therapeutic strategies might be developed.
- The mechanisms responsible for intestinal inflammation resolution needs further clarification. M2 macrophages accumulation in the intestinal microenvironment appears crucial. Identifying and studying environmental signals regulating phenotypes and functions of intestinal macrophages, such as AhR ligands, is essential in homeostasis and inflammation. Modulating monocytes to mature M2 macrophages transition through new identified molecules and pathways could promote remission of chronic states.

## Conclusions

GM has positioned itself as an element that greatly impacts both intestinal and systemic immunity. A better understanding of GM importance, its metabolites, interacting receptors, the transcriptional regulation metabolites exert, and eventual interactions between various metabolites, provide countless possibilities in the prevention and treatment of inflammatory pathologies in western societies (obesity, diabetes, hypertension, neurodegenerative disorders, among others). Intriguingly, there is an overlap with respect to the molecules regulating both intestinal and immune homeostasis and how the deletion of either one of these metabolites or its receptors is capable of affecting the proper functioning of the host. Alternatively, the crosstalk between bacterial metabolites and possible convergences on pathways of interest in the modulation of local and/or systemic immune responses, becomes an attractive area that could be exploited to maintain intestinal and systemic homeostasis.

Many questions need solving and many challenges remain, however, is clear that the manipulation of intestinal health and immunity through receptors, enzymes and transcription factors is a reality. Furthermore, the identification of important commensals in GM-host interactions and the production of bioactive molecules could open up a greater number of possibilities that will determine the importance of GM and its metabolites in the susceptibility and progression of inflammatory pathologies of the gastrointestinal tract.

Challenges exist in selecting and delivering the correct bacteria strains and/or bacteria clusters to promote bacteria abundance and metabolite loss in the patients. Faecal microbiota transplantation has been proposed as a new treatment, mainly for *Clostridium difficile* infection and Crohn’s disease. However, despite being effective in alleviating symptoms, this procedure has drawbacks associated with safety of the transplanted inoculum and the difficulty of standardizing their bacterial content. Therefore, rational design of standardized consortia of intestinal bacteria for the efficient and safe treatment of these pathologies represent an alternative to faecal microbiota transplantation; associated to production of different bacterial metabolites inhibiting inflammatory signaling, thus reducing inflammation. Alternatively, the promotion of healthy food consumption, rich in essential substrates in the synthesis of bacterial metabolites, as well as foods or food stuffs capable of modulating GM, could limit a wide range of inflammatory diseases prevalent nowadays.

## Author Contributions

NG wrote most of the review. PV and MH participated reviewing and critically correcting the manuscript, on manuscript structure and supervised the work. All authors contributed to the article and approved the submitted version.

## Funding

This work was funded by the National Agency for Research and Development (ANID)/Scholarship Program/DOCTORADO BECAS NACIONAL/2020 – 21200669, FONDECYT 1170648, Redes 180134 and FONDAP 15130011 Grants (MAH).

## Conflict of Interest

The authors declare that the research was conducted in the absence of any commercial or financial relationships that could be construed as a potential conflict of interest.

## References

[1] Yap YA Mariño E. An Insight Into the Intestinal Web of Mucosal Immunity, Microbiota, and Diet in Inflammation. Front Immunol (2018) 9(NOV):1–9. 10.3389/fimmu.2018.02617 30532751 PMC6266996

[2] Venegas DP De La Fuente MK Landskron G González MJ Quera R Dijkstra G. Short Chain Fatty Acids (Scfas)Mediated Gut Epithelial and Immune Regulation and its Relevance for Inflammatory Bowel Diseases. Front Immunol Front Media SA (2019) 10:277. 10.3389/fimmu.2019.00277 PMC6421268 30915065

[3] Zafeiropoulou K Nichols B Mackinder M Biskou O Rizou E Karanikolou A. Alterations in Intestinal Microbiota of Children With Celiac Disease At the Time of Diagnosis and on a Gluten-free Diet. Gastroenterology (2020) 159(6):2039–2051.e20. 10.1053/j.gastro.2020.08.007 32791131 PMC7773982

[4] Gordon JI. Honor Thy Gut Symbionts Redux. Sci (80- ) (2012) 336(6086):1251–3. 10.1126/science.1224686 22674326

[5] Gasaly N Riveros K Gotteland M. Fitoquímicos: Una Nueva Clase De Prebióticos/Phytochemicals: A New Class of Prebiotics. Rev Chil Nutr (2020) 47(2):317–27. 10.4067/S0717-75182020000200317

[6] Fujio-Vejar S Vasquez Y Morales P Magne F Vera-Wolf P Ugalde JA. The Gut Microbiota of Healthy Chilean Subjects Reveals a High Abundance of the Phylum Verrucomicrobia. Front Microbiol (2017) 8(JUN):1–11. 10.3389/fmicb.2017.01221 28713349 PMC5491548

[7] Nicholson JK Wilson ID. Understanding “Global” Systems Biology: Metabonomics and the Continuum of Metabolism. Nat Rev Drug Discov (2003) 2(8):668–76. 10.1038/nrd1157 12904817

[8] Zheng D Liwinski T Elinav E. Interaction Between Microbiota and Immunity in Health and Disease. Cell Res (2020) 30(6):492–506. 10.1038/s41422-020-0332-7 32433595 PMC7264227

[9] Hold GL. Gastrointestinal Microbiota and Colon Cancer. Dig Dis (2016) 34(3):244–50. 10.1159/000443358 27028619

[10] Anders HJ Andersen K Stecher B. The Intestinal Microbiota, a Leaky Gut, and Abnormal Immunity in Kidney Disease. Kidney Int (2013) 83(6):1010–6. 10.1038/ki.2012.440 23325079

[11] Blumberg R Powrie F. Microbiota, Disease, and Back to Health: A Metastable Journey. Sci Transl Med (2012) 4(137):137rv7. 10.1126/scitranslmed.3004184 PMC5020897 22674557

[12] Ludwig IS Broere F Manurung S Lambers TT van der Zee R van Eden W. Lactobacillus Rhamnosus GG-derived Soluble Mediators Modulate Adaptive Immune Cells. Front Immunol (2018) 9(JUL):1–6. 10.3389/fimmu.2018.01546 30042761 PMC6048560

[13] Thorburn AN Macia L Mackay CR. Diet, Metabolites, and “Western-Lifestyle” Inflammatory Diseases. Immun (2014) 40(6):833–42. 10.1016/j.immuni.2014.05.014 24950203

[14] Hamard A Sève B Le Floc’h N. Intestinal Development and Growth Performance of Early-Weaned Piglets Fed a Low-Threonine Diet. Animal (2007) 1(8):1134–42. 10.1017/S1751731107000560 22444859

[15] Mowat AMI. To Respond or Not to Respond - A Personal Perspective of Intestinal Tolerance. Nat Rev Immunol (2018) 18(6):405–15. 10.1038/s41577-018-0002-x 29491358

[16] Belkaid Y Naik S. Compartmentalized and Systemic Control of Tissue Immunity by Commensals. Nat Immunol (2013) 14(7):646–53. 10.1038/ni.2604 PMC3845005 23778791

[17] Shan M Gentile M Yeiser JR Walland AC Bornstein VU Chen K. Mucus Enhances Gut Homeostasis and Oral Tolerance by Delivering Immunoregulatory Signals. Sci (80- ) (2013) 342(6157):447–53. 10.1126/science.1237910 PMC4005805 24072822

[18] Groschwitz KR Hogan SP. Intestinal Barrier Function: Molecular Regulation and Disease Pathogenesis. J Allergy Clin Immunol (2009) 124(1):3–20. 10.1016/j.jaci.2009.05.038 19560575 PMC4266989

[19] Bansal T Alaniz RC Wood TK Jayaraman A. The Bacterial Signal Indole Increases Epithelial-Cell Tight-Junction Resistance and Attenuates Indicators of Inflammation. Proc Natl Acad Sci U S A (2010) 107(1):228–33. 10.1073/pnas.0906112107 PMC2806735 19966295

[20] Yan B Liu S Shi Y Liu N Chen L Wang X. Activation of AhR With Nuclear Ikkα Regulates Cancer Stem-Like Properties in the Occurrence of Radioresistance. Cell Death Dis (2018) 9(5):490. 10.1038/s41419-018-0542-9 29706625 PMC5924755

[21] Kim KS Hong SW Han D Yi J Jung J Yang BG. Dietary Antigens Limit Mucosal Immunity by Inducing Regulatory T Cells in the Small Intestine. Sci (80- ) (2016) 351(6275):858–63. 10.1126/science.aac5560 26822607

[22] Gálvez EJC Iljazovic A Gronow A Flavell R Strowig T. Shaping of Intestinal Microbiota in Nlrp6- and Rag2-Deficient Mice Depends on Community Structure. Cell Rep (2017) 21(13):3914–26. 10.1016/j.celrep.2017.12.027 29281837

[23] Tanoue T Morita S Plichta DR Skelly AN Suda W Sugiura Y. A Defined Commensal Consortium Elicits CD8 T Cells and Anti-Cancer Immunity. Nature (2019) 565(7741):600–5. 10.1038/s41586-019-0878-z 30675064

[24] Makki K Deehan EC Walter J Bäckhed F. The Impact of Dietary Fiber on Gut Microbiota in Host Health and Disease. Cell Host Microbe (2018) 23(6):705–15. 10.1016/j.chom.2018.05.012 29902436

[25] Soliman GA. Dietary Fiber, Atherosclerosis, and Cardiovascular Disease. Nutrients (2019) 11(5):1155. 10.3390/nu11051155 PMC6566984 31126110

[26] Slavin J. Fiber and Prebiotics: Mechanisms and Health Benefits. Nutrients (2013) 5(4):1417–35. 10.3390/nu5041417 PMC3705355 23609775

[27] Reichardt N Vollmer M Holtrop G Farquharson FM Wefers D Bunzel M. Specific Substrate-Driven Changes in Human Faecal Microbiota Composition Contrast With Functional Redundancy in Short-Chain Fatty Acid Production. ISME J (2018) 12(2):610–22. 10.1038/ismej.2017.196 PMC5776475 29192904

[28] Wang M Wichienchot S He X Fu X Huang Q Zhang B. In Vitro Colonic Fermentation of Dietary Fibers: Fermentation Rate, Short-Chain Fatty Acid Production and Changes in Microbiota. Trends Food Sci Technol (2019) 88(March):1–9. 10.1016/j.tifs.2019.03.005

[29] Saura-Calixto F. Dietary Fiber as a Carrier of Dietary Antioxidants: An Essential Physiological Function. J Agric Food Chem (2011) 59(1):43–9. 10.1021/jf1036596 21142013

[30] Karlsson FH Fåk F Nookaew I Tremaroli V Fagerberg B Petranovic D. Symptomatic Atherosclerosis is Associated With an Altered Gut Metagenome. Nat Commun (2012) 3:1245. 10.1038/ncomms2266 23212374 PMC3538954

[31] Fu J Bonder MJ Cenit MC Tigchelaar EF Maatman A Dekens JAM. The Gut Microbiome Contributes to a Substantial Proportion of the Variation in Blood Lipids. Circ Res (2015) 117(9):817–24. 10.1161/CIRCRESAHA.115.306807 PMC4596485 26358192

[32] Fardet A. New Hypotheses for the Health-Protective Mechanisms of Whole-Grain Cereals: What is Beyond Fibre? Nutr Res Rev (2010) 23(1):65–134. 10.1017/S0954422410000041 20565994

[33] Aune D Chan DSM Lau R Vieira R Greenwood DC Kampman E. Dietary Fibre, Whole Grains, and Risk of Colorectal Cancer: Systematic Review and Dose-Response Meta-Analysis of Prospective Studies. BMJ (2011) 343(7833):1082. 10.1136/bmj.d6617 PMC3213242 22074852

[34] Reynolds A Mann J Cummings J Winter N Mete E Te Morenga L. Carbohydrate Quality and Human Health: A Series of Systematic Reviews and Meta-Analyses. Lancet (2019) 393(10170):434–45. 10.1016/S0140-6736(18)31809-9 30638909

[35] Sonnenburg ED Sonnenburg JL. Starving Our Microbial Self: The Deleterious Consequences of a Diet Deficient in Microbiota-Accessible Carbohydrates. Cell Metab (2014) 20(5):779–86. 10.1016/j.cmet.2014.07.003 PMC4896489 25156449

[36] Mu Q Kirby J Reilly CM Luo XM. Leaky Gut as a Danger Signal for Autoimmune Diseases. Front Immunol (2017) 8(MAY):1–10. 10.3389/fimmu.2017.00598 28588585 PMC5440529

[37] McGuckin MA Eri R Simms LA Florin THJ Radford-Smith G. Intestinal Barrier Dysfunction in Inflammatory Bowel Diseases. Inflammation Bowel Dis (2009) 15(1):100–13. 10.1002/ibd.20539 18623167

[38] Beukema M Faas MM de Vos P. The Effects of Different Dietary Fiber Pectin Structures on the Gastrointestinal Immune Barrier: Impact Via Gut Microbiota and Direct Effects on Immune Cells. Exp Mol Med (2020) 52:1364–76. 10.1038/s12276-020-0449-2 PMC8080816 32908213

[39] Breton J Plé C Guerin-Deremaux L Pot B Lefranc-Millot C Wils D. Intrinsic Immunomodulatory Effects of Low-Digestible Carbohydrates Selectively Extend Their Anti-Inflammatory Prebiotic Potentials. BioMed Res Int (2015) 2015:162398. 10.1155/2015/162398 25977916 PMC4419225

[40] Sanders ME Merenstein DJ Reid G Gibson GR Rastall RA. Probiotics and Prebiotics in Intestinal Health and Disease: From Biology to the Clinic. Nat Rev Gastroenterol Hepatol (2019) 16(10):605–16. 10.1038/s41575-019-0173-3 31296969

[41] Carlson JL Erickson JM Hess JM Gould TJ Slavin JL. Prebiotic Dietary Fiber and Gut Health: Comparing the In Vitro Fermentations of Beta-Glucan, Inulin and Xylooligosaccharide. Nutrients (2017) 9(12):1361. 10.3390/nu9121361 PMC5748811 29244718

[42] Whisner CM Castillo LF. Prebiotics, Bone and Mineral Metabolism. Calcif Tissue Int (2018) 102(4):443–79. 10.1007/s00223-017-0339-3 PMC5851694 29079996

[43] Alfa MJ Strang D Tappia PS Graham M Van Domselaar G Forbes JD. A Randomized Trial to Determine the Impact of a Digestion Resistant Starch Composition on the Gut Microbiome in Older and Mid-Age Adults. Clin Nutr (2018) 37(3):797–807. 10.1016/j.clnu.2017.03.025 28410921

[44] Louis P Flint HJ. Diversity, Metabolism and Microbial Ecology of Butyrate-Producing Bacteria From the Human Large Intestine. FEMS Microbiol Lett (2009) 294(1):1–8. 10.1111/j.1574-6968.2009.01514.x 19222573

[45] Louis P Flint HJ. Formation of Propionate and Butyrate by the Human Colonic Microbiota. Environ Microbiol (2017) 19(1):29–41. 10.1111/1462-2920.13589 27928878

[46] Vital M Howe AC Tiedje JM. Revealing the Bacterial Butyrate Synthesis Pathways by Analyzing (Meta)Genomic Data. MBio (2014) 5(2):1–11. 10.1128/mBio.00889-14 PMC3994512 24757212

[47] Rivière A Selak M Lantin D Leroy F De Vuyst L. Bifidobacteria and Butyrate-Producing Colon Bacteria: Importance and Strategies for Their Stimulation in the Human Gut. Front Microbiol (2016) 7:979. 10.3389/fmicb.2016.00979 27446020 PMC4923077

[48] Martinez-Gutierrez F Ratering S Juárez-Flores B Godinez-Hernandez C Geissler-Plaum R Prell F. Potential Use of Agave Salmiana as a Prebiotic That Stimulates the Growth of Probiotic Bacteria. Lwt (2017) 84:151–9. 10.1016/j.lwt.2017.05.044

[49] Derrien M Vaughan EE Plugge CM de Vos WM. Akkermansia Municiphila Gen. Nov., Sp. Nov., a Human Intestinal Mucin-Degrading Bacterium. Int J Syst Evol Microbiol (2004) 54(5):1469–76. 10.1099/ijs.0.02873-0 15388697

[50] Kellow NJ Coughlan MT Reid CM. Metabolic Benefits of Dietary Prebiotics in Human Subjects: A Systematic Review of Randomised Controlled Trials. Br J Nutr Cambridge Univ Press; (2014) 111:1147–61. 10.1017/S0007114513003607 24230488

[51] Vinolo MAR Rodrigues HG Nachbar RT Curi R. Regulation of Inflammation by Short Chain Fatty Acids. Nutrients MDPI AG; (2011) 3:858–76. 10.3390/nu3100858 PMC3257741 22254083

[52] Peng L He Z Chen W Holzman IR Lin J. Effects of Butyrate on Intestinal Barrier Function in a Caco-2 Cell Monolayer Model of Intestinal Barrier. Pediatr Res (2007) 61(1):37–41. 10.1203/01.pdr.0000250014.92242.f3 17211138

[53] Peng L Li ZR Green RS Holzman IR Lin J. Butyrate Enhances the Intestinal Barrier by Facilitating Tight Junction Assembly Via Activation of AMP-activated Protein Kinase in Caco-2 Cell Monolayers. J Nutr (2009) 139(9):1619–25. 10.3945/jn.109.104638 PMC2728689 19625695

[54] Nielsen DSG Jensen BB Theil PK Nielsen TS Knudsen KEB Purup S. Effect of Butyrate and Fermentation Products on Epithelial Integrity in a Mucus-Secreting Human Colon Cell Line. J Funct Foods (2018) 40(October 2017):9–17. 10.1016/j.jff.2017.10.023

[55] Yan H Ajuwon KM. Butyrate Modifies Intestinal Barrier Function in IPEC-J2 Cells Through a Selective Upregulation of Tight Junction Proteins and Activation of the Akt Signaling Pathway. PloS One (2017) 12(6):1–20. 10.1371/journal.pone.0179586 PMC5487041 28654658

[56] Nafday SM Chen W Peng L Babyatsky MW Holzman IR Lin J. Short-Chain Fatty Acids Induce Colonic Mucosal Injury in Rats With Various Postnatal Ages. Pediatr Res (2005) 57(2):201–4. 10.1203/01.PDR.0000150721.83224.89 15611351

[57] Carding S Verbeke K Vipond DT Corfe BM Owen LJ. Dysbiosis of the Gut Microbiota in Disease. Microb Ecol Heal Dis (2015) 26:26191. 10.3402/mehd.v26.26191 PMC4315779 25651997

[58] Beukema M Faas MM de Vos P. The Effects of Different Dietary Fiber Pectin Structures on the Gastrointestinal Immune Barrier: Impact Via Gut Microbiota and Direct Effects on Immune Cells. Exp Mol Med (2020) 52:1364–76. 10.1038/s12276-020-0449-2 PMC8080816 32908213

[59] Böcker U Böcker U Nebe T Herweck F Holt L Panja A. Immunomodulatory Effects of Butyrate on IEC Butyrate Modulates Intestinal Epithelial Cell-Mediated Neutrophil Migration. Clin Exp Immunol (2003) 131(1):53–60. 10.1046/j.1365-2249.2003.02056.x PMC1808611 12519386

[60] Asarat M Vasiljevic T Apostolopoulos V Donkor O. Short-Chain Fatty Acids Regulate Secretion of IL-8 From Human Intestinal Epithelial Cell Lines In Vitro. Immunol Invest (2015) 44(7):678–93. 10.3109/08820139.2015.1085389 26436853

[61] Malago JJ Koninkx JFJG Tooten PCJ Van Liere EA Van Dijk JE. Anti-Inflammatory Properties of Heat Shock Protein 70 and Butyrate on Salmonella-induced Interleukin-8 Secretion in Enterocyte-Like Caco-2 Cells. Clin Exp Immunol (2005) 141(1):62–71. 10.1111/j.1365-2249.2005.02810.x 15958071 PMC1809404

[62] Donohoe DR Collins LB Wali A Bigler R Sun W Bultman SJ. The Warburg Effect Dictates the Mechanism of Butyrate-Mediated Histone Acetylation and Cell Proliferation. Mol Cell (2012) 48(4):612–26. 10.1016/j.molcel.2012.08.033 PMC3513569 23063526

[63] Liu J Zhu H Li B Lee C Alganabi M Zheng S. Beneficial Effects of Butyrate in Intestinal Injury. J Pediatr Surg (2020) 55(6):1088–93. 10.1016/j.jpedsurg.2020.02.036 32234318

[64] Mariadason JM Velcich A Wilson AJ Augenlicht LH Gibson PR. Resistance to Butyrate-Induced Cell Differentiation and Apoptosis During Spontaneous Caco-2 Cell Differentiation. Gastroenterology (2001) 120(4):889–99. 10.1053/gast.2001.22472 11231943

[65] Macfarlane S Macfarlane GT. Regulation of Short-Chain Fatty Acid Production. Proc Nutr Soc (2003) 62(1):67–72. 10.1079/PNS2002207 12740060

[66] Borthakur A Priyamvada S Kumar A Natarajan AA Gill RK Alrefai WA. A Novel Nutrient Sensing Mechanism Underlies Substrate-Induced Regulation of Monocarboxylate Transporter-1. Am J Physiol Gastrointest Liver Physiol (2012) 303:1126–33. 10.1152/ajpgi.00308.2012 PMC3517653 22982338

[67] Karaki SI Mitsui R Hayashi H Kato I Sugiya H Iwanaga T. Short-Chain Fatty Acid Receptor, GPR43, is Expressed by Enteroendocrine Cells and Mucosal Mast Cells in Rat Intestine. Cell Tissue Res (2006) 324(3):353–60. 10.1007/s00441-005-0140-x 16453106

[68] Thangaraju M Cresci GA Liu K Ananth S Gnanaprakasam JP Browning DD. GPR109A is a G-protein-coupled Receptor for the Bacterial Fermentation Product Butyrate and Functions as a Tumor Suppressor in Colon. Cancer Res (2009) 69(7):2826–32. 10.1158/0008-5472.CAN-08-4466 PMC3747834 19276343

[69] Thibault R De Coppet P Daly K Bourreille A Cuff M Bonnet C. Down-Regulation of the Monocarboxylate Transporter 1 Is Involved in Butyrate Deficiency During Intestinal Inflammation. Gastroenterology (2007) 133(6):1916–27. 10.1053/j.gastro.2007.08.041 18054563

[70] Nancey S Blanvillain E Parmentier B Flourié B Bayet C Bienvenu J. Infliximab Treatment Does Not Induce Organ-specific or Nonorgan-specific Autoantibodies Other Than Antinuclear and Anti-Double-Stranded DNA Autoantibodies in Crohn’s Disease. Inflamm Bowel Dis (2005) 11(11):986–91. 10.1097/01.mib.0000186408.07769.78 16239844

[71] De Preter V Bulteel V Suenaert P Geboes KP De Hertogh G Luypaerts A. Pouchitis, Similar to Active Ulcerative Colitis, is Associated With Impaired Butyrate Oxidation by Intestinal Mucosa. Inflamm Bowel Dis (2009) 15(3):335–40. 10.1002/ibd.20768 18942762

[72] Roediger WEW. The Starved Colon Diminished Mucosal Diminished Absorption, and Colitis Nutrition. Dis Colon Rectum (1990) 33(10):858–62. 10.1007/BF02051922 2209275

[73] Shao BZ Xu ZQ Han BZ Su DF Liu C. NLRP3 Inflammasome and its Inhibitors: A Review. Front Pharmacol (2015) 6(NOV):1–9. 10.3389/fphar.2015.00262 26594174 PMC4633676

[74] Mangan MSJ Olhava EJ Roush WR Seidel HM Glick GD Latz E. Targeting the NLRP3 Inflammasome in Inflammatory Diseases. Nat Rev Drug Discovery (2018) 17(8):588–606. 10.1038/nrd.2018.97 30026524

[75] Rabeony H Pohin M Vasseur P Petit-Paris I Jégou JF Favot L. IMQ-Induced Skin Inflammation in Mice is Dependent on IL-1R1 and MyD88 Signaling But Independent of the NLRP3 Inflammasome. Eur J Immunol (2015) 45(10):2847–57. 10.1002/eji.201445215 26147228

[76] Kespohl M Vachharajani N Luu M Harb H Pautz S Wolff S. The Microbial Metabolite Butyrate Induces Expression of Th1- Associated Factors in Cd4+ T Cells. Front Immunol (2017) 8:1036. 10.3389/fimmu.2017.01036 28894447 PMC5581317

[77] Kaisar MMM Pelgrom LR van der Ham AJ Yazdanbakhsh M Everts B. Butyrate Conditions Human Dendritic Cells to Prime Type 1 Regulatory T Cells Via Both Histone Deacetylase Inhibition and G Protein-Coupled Receptor 109A Signaling. Front Immunol (2017) 8(OCT):1–14. 10.3389/fimmu.2017.01429 29163504 PMC5670331

[78] Singh N Gurav A Sivaprakasam S Brady E Padia R Shi H. Activation of Gpr109a, Receptor for Niacin and the Commensal Metabolite Butyrate, Suppresses Colonic Inflammation and Carcinogenesis. Immunity (2014) 40(1):128–39. 10.1016/j.immuni.2013.12.007 PMC4305274 24412617

[79] Nastasi C Fredholm S Willerslev-Olsen A Hansen M Bonefeld CM Geisler C. Butyrate and Propionate Inhibit Antigen-Specific CD8+ T Cell Activation by Suppressing IL-12 Production by Antigen-Presenting Cells. Sci Rep (2017) 7(1):1–10. 10.1038/s41598-017-15099-w 29109552 PMC5673935

[80] Liu H Wang J He T Becker S Zhang G Li D. Butyrate: A Double-Edged Sword for Health? Adv Nutr (2018) 9(1):21–9. 10.1093/advances/nmx009 PMC6333934 29438462

[81] Nowarski R Jackson R Gagliani N De Zoete MR Palm NW Bailis W. Epithelial IL-18 Equilibrium Controls Barrier Function in Colitis. Cell (2015) 163(6):1444–56. 10.1016/j.cell.2015.10.072 PMC4943028 26638073

[82] Sokol H dicte Pigneur B Watterlot L Lakhdari O Bermú dez-Humará LG Gratadoux J-J. Faecalibacterium Prausnitzii is an Anti-Inflammatory Commensal Bacterium Identified by Gut Microbiota Analysis of Crohn Disease Patients. Proc Natl Acad Sci USA (2008) 105(43):16731–36. 10.1073/pnas.0804812105 PMC2575488 18936492

[83] Wang W Chen L Zhou R Wang X Song L Huang S. Increased Proportions of Bifidobacterium and the Lactobacillus Group and Loss of Butyrate-Producing Bacteria in Inflammatory Bowel Disease. J Clin Microbiol (2014) 52(2):398–406. 10.1128/JCM.01500-13 24478468 PMC3911339

[84] Takahashi K Nishida A Fujimoto T Fujii M Shioya M Imaeda H. Reduced Abundance of Butyrate-Producing Bacteria Species in the Fecal Microbial Community in Crohn’s Disease. Digestion (2016) 93(1):59–65. 10.1159/000441768 26789999

[85] Frank DN St Amand AL Feldman RA Boedeker EC Harpaz N Pace NR. Molecular-Phylogenetic Characterization of Microbial Community Imbalances in Human Inflammatory Bowel Diseases. (2007) 104(34):13780–5. 10.1073/pnas.0706625104 PMC1959459 17699621

[86] Sartor RB. The Intestinal Microbiota in Inflammatory Bowel Diseases. Nestle Nutrition Institute Workshop Series (2014) 79:29–39. 10.1159/000360674 25227293

[87] Laserna-Mendieta EJ Clooney AG Carretero-Gomez JF Moran C Sheehan D Nolan JA. Determinants of Reduced Genetic Capacity for Butyrate Synthesis by the Gut Microbiome in Crohn’s Disease and Ulcerative Colitis. J Crohn’s Colitis (2018) 12(2):204–16. 10.1093/ecco-jcc/jjx137 29373727

[88] Niccolai E Baldi S Ricci F Russo E Nannini G Menicatti M. Evaluation and Comparison of Short Chain Fatty Acids Composition in Gut Diseases. World J Gastroenterol World J Gastroenterol (2019) 25(36):5403–577. 10.3748/wjg.v25.i36.5543 PMC6767983 31576099

[89] Drabińska N Jarocka-Cyrta E Markiewicz LH Krupa-Kozak U. The Effect of Oligofructose-Enriched Inulin on Faecal Bacterial Counts and Microbiota-Associated Characteristics in Celiac Disease Children Following a Gluten-Free Diet: Results of a Randomized, Placebo-Controlled Trial. Nutrients (2018) 10(2):1–11. 10.3390/nu10020201 PMC5852777 29439526

[90] Lührs H Gerke T Müller JG Melcher R Schauber J Boxberger F. Butyrate Inhibits NF-µb Activation in Lamina Propria Macrophages of Patients With Ulcerative Colitis. Scand J Gastroenterol (2002) 37(4):458–66. 10.1080/003655202317316105 11989838

[91] Breuer RI Soergel KH Lashner BA Christ ML Hanauer SB Vanagunas A. Short Chain Fatty Acid Rectal Irrigation for Left-Sided Ulcerative Colitis: A Randomised, Placebo Controlled Trial. Gut (1997) 40(4):485–91. 10.1136/gut.40.4.485 PMC1027123 9176076

[92] Hamer HM Jonkers DMAE Vanhoutvin SALW Troost FJ Rijkers G de Bruïne A. Effect of Butyrate Enemas on Inflammation and Antioxidant Status in the Colonic Mucosa of Patients With Ulcerative Colitis in Remission. Clin Nutr (2010) 29(6):738–44. 10.1016/j.clnu.2010.04.002 20471725

[93] Gaudier E Jarry A Blottière HM De Coppet P Buisine MP Aubert JP. Butyrate Specifically Modulates MUC Gene Expression in Intestinal Epithelial Goblet Cells Deprived of Glucose. Am J Physiol - Gastrointest Liver Physiol (2004) 287(6 50-6):1168–74. 10.1152/ajpgi.00219.2004 15308471

[94] Ishisono K Yabe T Kitaguchi K. Citrus Pectin Attenuates Endotoxin Shock Via Suppression of Toll-like Receptor Signaling in Peyer’s Patch Myeloid Cells. J Nutr Biochem (2017) 50:38–45. 10.1016/j.jnutbio.2017.07.016 29031241

[95] Liu L Li YH Niu YB Sun Y Guo ZJ Li Q. An Apple Oligogalactan Prevents Against Inflammation and Carcinogenesis by Targeting LPS/TLR4/NF-κb Pathway in a Mouse Model of Colitis-Associated Colon Cancer. Carcinogenesis (2010) 31(10):1822–32. 10.1093/carcin/bgq070 20400476

[96] Bermudez-Brito M Rösch C Schols HA Faas MM de Vos P. Resistant Starches Differentially Stimulate Toll-like Receptors and Attenuate Proinflammatory Cytokines in Dendritic Cells by Modulation of Intestinal Epithelial Cells. Mol Nutr Food Res (2015) 59(9):1814–26. 10.1002/mnfr.201500148 26015170

[97] Beukema M Jermendi É Schols HA de Vos P. The Influence of Calcium on Pectin’s Impact on TLR2 Signalling. Food Funct (2020) 11(9):7427–32. 10.1039/D0FO01703E 32902547

[98] Vogt L Ramasamy U Meyer D Pullens G Venema K Faas MM. Immune Modulation by Different Types of β2→1-Fructans Is Toll-Like Receptor Dependent. PloS One (2013) 8(7):1–12. 10.1371/journal.pone.0068367 PMC3702581 23861894

[99] Vogt LM Meyer D Pullens G Faas MM Venema K Ramasamy U. Toll-Like Receptor 2 Activation by β2→1-Fructans Protects Barrier Function of t84 Human Intestinal Epithelial Cells in a Chain Length-Dependent Manner. J Nutr (2014) 144(7):1002–8. 10.3945/jn.114.191643 24790027

[100] Chen H Mao X He J Yu B Huang Z Yu J. Dietary Fibre Affects Intestinal Mucosal Barrier Function and Regulates Intestinal Bacteria in Weaning Piglets. Br J Nutr (2013) 110(10):1837–48. 10.1017/S0007114513001293 23656640

[101] Sabater C Molina-Tijeras JA Vezza T Corzo N Montilla A Utrilla P. Intestinal Anti-Inflammatory Effects of Artichoke Pectin and Modified Pectin Fractions in the Dextran Sulfate Sodium Model of Mice Colitis. Artificial Neural Network Modelling of Inflammatory Markers. Food Funct (2019) 10(12):7793–805. 10.1039/C9FO02221J 31781703

[102] Van Hung T Suzuki T. Guar Gum Fiber Increases Suppressor of Cytokine Signaling-1 Expression Via Toll-Like Receptor 2 and Dectin-1 Pathways, Regulating Inflammatory Response in Small Intestinal Epithelial Cells. Mol Nutr Food Res (2017) 61:1–39 p. 10.1002/mnfr.201700048 28608623

[103] Kumalasari ID Nishi K Putra ABN Sugahara T. Activation of Macrophages Stimulated by the Bengkoang Fiber Extract Through Toll-Like Receptor 4. Food Funct (2014) 5(7):1403–8. 10.1039/c3fo60360a 24770453

[104] Lépine A de Vos P. Synbiotic Effects of the Dietary Fiber Long-Chain Inulin and Probiotic Lactobacillus Acidophilus W37 can be Caused by Direct, Synergistic Stimulation of Immune Toll-Like Receptors and Dendritic Cells. Mol Nutr Food Res (2018) 62(15):1–28. 10.1002/mnfr.201800251 PMC6099370 29902355

[105] Wu RY Abdullah M Määttänen P Pilar AVC Scruten E Johnson-Henry KC. Protein Kinase Cς Signaling is Required for Dietary Prebiotic-Induced Strengthening of Intestinal Epithelial Barrier Function. Sci Rep (2017) 7(September 2016):1–10. 10.1038/srep40820 28098206 PMC5241689

[106] Liu E Lee H-S Aronsson CA Hagopian WA Koletzko S Rewers MJ. Risk of Pediatric Celiac Disease According to HLA Haplotype and Country. N Engl J Med (2014) 371(1):42–9. 10.1056/NEJMoa1313977 PMC4163840 24988556

[107] Lund-Blix NA Tapia G Mårild K Brantsæter AL Eggesbø M Mandal S. Maternal Fibre and Gluten Intake During Pregnancy and Risk of Childhood Celiac Disease: The MoBa Study. Sci Rep (2020) 10(1):1–9. 10.1038/s41598-020-73244-4 33009438 PMC7532434

[108] Sciacchitano S Lavra L Morgante A Ulivieri A Magi F De Francesco GP. Galectin-3: One Molecule for an Alphabet of Diseases, From A to Z. Int J Mol Sci (2018) 19(2):379. 10.3390/ijms19020379 PMC5855601 29373564

[109] Díaz-Alvarez L Ortega E. The Many Roles of Galectin-3, a Multifaceted Molecule, in Innate Immune Responses Against Pathogens. Mediators Inflammation (2017) 2017:9–12. 10.1155/2017/9247574 PMC5457773 28607536

[110] Gao X Zhi Y Sun L Peng X Zhang T Xue H. The Inhibitory Effects of a Rhamnogalacturonan I (Rg-I) Domain From Ginseng Pectin on Galectin-3 and its Structure-Activity Relationship. J Biol Chem (2013) 288(47):33953–65. 10.1074/jbc.M113.482315 PMC3837135 24100038

[111] De Aguiar Vallim TQ Tarling EJ Edwards PA. Pleiotropic Roles of Bile Acids in Metabolism. Cell Metab (2013) 17(5):657–69. 10.1016/j.cmet.2013.03.013 PMC3654004 23602448

[112] Sayin SI Wahlström A Felin J Jäntti S Marschall HU Bamberg K. Gut Microbiota Regulates Bile Acid Metabolism by Reducing the Levels of Tauro-Beta-Muricholic Acid, a Naturally Occurring FXR Antagonist. Cell Metab (2013) 17(2):225–35. 10.1016/j.cmet.2013.01.003 23395169

[113] Ma H Patti ME Endocrinologist A. Bile Acids, Obesity, and the Metabolic Syndrome. Best Pract Res Clin Gastroenterol (2014) 28(4):573–83. 10.1016/j.bpg.2014.07.004 PMC4159616 25194176

[114] Long SL Gahan CGM Joyce SA. Interactions Between Gut Bacteria and Bile in Health and Disease. Mol Aspects Med (2017) 56:54–65. 10.1016/j.mam.2017.06.002 28602676

[115] Gérard P. Metabolism of Cholesterol and Bile Acids by the Gut Microbiota. Pathogens (2013) 3(1):14–24. 10.3390/pathogens3010014 25437605 PMC4235735

[116] Ridlon JM Kang DJ Hylemon PB. Bile Salt Biotransformations by Human Intestinal Bacteria. J Lipid Res (2006) 47(2):241–59. 10.1194/jlr.R500013-JLR200 16299351

[117] Jones BV Begley M Hill C Gahan CGM Marchesi JR. Functional and Comparative Metagenomic Analysis of Bile Salt Hydrolase Activity in the Human Gut Microbiome. Proc Natl Acad Sci U S A (2008) 105(36):13580–5. 10.1073/pnas.0804437105 PMC2533232 18757757

[118] Duboc H Rajca S Rainteau D Benarous D Maubert MA Quervain E. Connecting Dysbiosis, Bile-Acid Dysmetabolism and Gut Inflammation in Inflammatory Bowel Diseases. Gut (2013) 62(4):531–9. 10.1136/gutjnl-2012-302578 22993202

[119] Ridlon JM Kang DJ Hylemon PB. Isolation and Characterization of a Bile Acid Inducible 7α-Dehydroxylating Operon in Clostridium Hylemonae TN271. Anaerobe (2010) 16(2):137–46. 10.1016/j.anaerobe.2009.05.004 PMC6262846 19464381

[120] Ridlon JM Harris SC Bhowmik S Kang DJ Hylemon PB. Consequences of Bile Salt Biotransformations by Intestinal Bacteria. Gut Microbes (2016) 7(1):22–39. 10.1080/19490976.2015.1127483 26939849 PMC4856454

[121] Jia W Xie G Jia W. Bile Acid–Microbiota Crosstalk in Gastrointestinal Inflammation and Carcinogenesis. Nat Rev Gastroenterol Hepatol (2018) 15(2):111–28. 10.1038/nrgastro.2017.119 PMC5899973 29018272

[122] Kundu S Kumar S Bajaj A. Cross-Talk Between Bile Acids and Gastrointestinal Tract for Progression and Development of Cancer and its Therapeutic Implications. IUBMB Life (2015) 67(7):514–23. 10.1002/iub.1399 26177921

[123] Fiorucci S Distrutti E. Bile Acid-Activated Receptors, Intestinal Microbiota, and the Treatment of Metabolic Disorders. Trends Mol Med (2015) 21(11):702–14. 10.1016/j.molmed.2015.09.001 26481828

[124] Maruyama T Miyamoto Y Nakamura T Tamai Y Okada H Sugiyama E. Identification of Membrane-Type Receptor for Bile Acids (M-BAR). Biochem Biophys Res Commun (2002) 298(5):714–9. 10.1016/S0006-291X(02)02550-0 12419312

[125] Vavassori P Mencarelli A Renga B Distrutti E Fiorucci S. The Bile Acid Receptor Fxr Is a Modulator of Intestinal Innate Immunity. J Immunol (2009) 183(10):6251–61. 10.4049/jimmunol.0803978 19864602

[126] Mencarelli A Renga B Migliorati M Cipriani S Distrutti E Santucci L. The Bile Acid Sensor Farnesoid X Receptor Is a Modulator of Liver Immunity in a Rodent Model of Acute Hepatitis. J Immunol (2009) 183(10):6657–66. 10.4049/jimmunol.0901347 19880446

[127] Cipriani S Mencarelli A Chini MG Distrutti E Renga B Bifulco G. The Bile Acid Receptor GPBAR-1 (TGR5) Modulates Integrity of Intestinal Barrier and Immune Response to Experimental Colitis. PloS One (2011) 6(10):e25637. 10.1371/journal.pone.0025637 22046243 PMC3203117

[128] Lu TT Makishima M Repa JJ Schoonjans K Kerr TA Auwerx J. Molecular Basis for Feedback Regulation of Bile Acid Synthesis by Nuclear Receptos. Mol Cell (2000) 6(3):507–15. 10.1016/S1097-2765(00)00050-2 11030331

[129] Cariello M Piccinin E Garcia-Irigoyen O Sabbà C Moschetta A. Nuclear Receptor FXR, Bile Acids and Liver Damage: Introducing the Progressive Familial Intrahepatic Cholestasis With FXR Mutations. Biochim Biophys Acta - Mol Basis Dis (2018) 1864(4):1308–18. 10.1016/j.bbadis.2017.09.019 28965883

[130] Chávez-Talavera O Tailleux A Lefebvre P Staels B. Bile Acid Control of Metabolism and Inflammation in Obesity, Type 2 Diabetes, Dyslipidemia, and Nonalcoholic Fatty Liver Disease. Gastroenterol Elsevier Inc (2017) 152:1679–94.e3. 10.1053/j.gastro.2017.01.055 28214524

[131] Das P Marcisauskas S Ji BNJ. Metagenomic Analysis of Bile Salt Biotransformation in the Human Gut Microbiome. BMC Genomics (2019) 20(517):517. 10.1186/s12864-019-5899-3 31234773 PMC6591925

[132] Heinken A Ravcheev DA Baldini F Heirendt L Fleming RMT Thiele I. Systematic Assessment of Secondary Bile Acid Metabolism in Gut Microbes Reveals Distinct Metabolic Capabilities in Inflammatory Bowel Disease. Microbiome (2019) 7(1):1–18. 10.1186/s40168-019-0689-3 31092280 PMC6521386

[133] Carr RM Reid AE. Fxr Agonists as Therapeutic Agents for Non-alcoholic Fatty Liver Disease. Curr Atheroscler Rep (2015) 17(4):500. 10.1007/s11883-015-0500-2 25690590

[134] Theriot CM Bowman AA Young B. Difficile Spore Germination and Outgrowth in the Large Intestine. Am Soc Microbiol (2016) 1(1):1–16. 10.1128/mSphere.00045-15 PMC4863611 27239562

[135] Modica S Murzilli S Salvatore L Schmidt DR Moschetta A. Nuclear Bile Acid Receptor FXR Protects Against Intestinal Tumorigenesis. Cancer Res (2008) 68(23):9589–94. 10.1158/0008-5472.CAN-08-1791 19047134

[136] Inagaki T Moschetta A Lee YK Peng L Zhao G Downes M. Regulation of Antibacterial Defense in the Small Intestine by the Nuclear Bile Acid Receptor. Proc Natl Acad Sci USA (2006) 103(10):3920–5. 10.1073/pnas.0509592103 PMC1450165 16473946

[137] Gadaleta RM Van Erpecum KJ Oldenburg B Willemsen ECL Renooij W Murzilli S. Farnesoid X Receptor Activation Inhibits Inflammation and Preserves the Intestinal Barrier in Inflammatory Bowel Disease. Gut (2011) 60(4):463–72. 10.1136/gut.2010.212159 21242261

[138] Campbell C McKenney PT Konstantinovsky D Isaeva OI Schizas M Verter J. Bacterial Metabolism of Bile Acids Promotes Generation of Peripheral Regulatory T Cells. Nature (2020) 581(7809):475–9. 10.1038/s41586-020-2193-0 PMC7540721 32461639

[139] Watanabe M Houten SM Mataki C Christoffolete MA Kim BW Sato H. Bile Acids Induce Energy Expenditure by Promoting Intracellular Thyroid Hormone Activation. Nature (2006) 439(7075):484–9. 10.1038/nature04330 16400329

[140] Jia Et Liu Z Pan M Lu Jf Ge Q. Regulation of Bile Acid Metabolism-Related Signaling Pathways by Gut Microbiota in Diseases. J Zhejiang Univ Sci B (2019) 20(10):781–92. 10.1631/jzus.B1900073 PMC6751489 31489798

[141] Biagioli M Carino A Cipriani S Francisci D Marchianò S Scarpelli P. The Bile Acid Receptor Gpbar1 Regulates the M1/M2 Phenotype of Intestinal Macrophages and Activation of GPBAR1 Rescues Mice From Murine Colitis. J Immunol (2017) 199(2):718–33. 10.4049/jimmunol.1700183 28607110

[142] Zundler S Neurath MF. Novel Insights Into the Mechanisms of Gut Homing and Antiadhesion Therapies in Inflammatory Bowel Diseases. Inflammation Bowel Dis (2017) 23(4):617–27. 10.1097/MIB.0000000000001067 28296823

[143] Sturgeon C Fasano A. Zonulin A. Regulator of Epithelial and Endothelial Barrier Functions, and Its Involvement in Chronic Inflammatory Diseases. Tissue Barriers (2016) 4(4):e1251384. 10.1080/21688370.2016.1251384 28123927 PMC5214347

[144] Schneider KM Albers S Trautwein C. Role of Bile Acids in the Gut-Liver Axis. J Hepatol (2018) 68(5):1083–5. 10.1016/j.jhep.2017.11.025 29519549

[145] Hoffmanová I Sánchez D Tučková L Tlaskalová-Hogenová H. Celiac Disease and Liver Disorders: From Putative Pathogenesis to Clinical Implications. Nutrients (2018) 10(7):1–17. 10.3390/nu10070892 PMC6073476 30002342

[146] Deutschmann K Reich M Klindt C Dröge C Spomer L Häussinger D. Bile Acid Receptors in the Biliary Tree: TGR5 in Physiology and Disease. Biochim Biophys Acta - Mol Basis Dis (2018) 1864(4):1319–25. 10.1016/j.bbadis.2017.08.021 28844960

[147] Agus A Planchais J Sokol H. Gut Microbiota Regulation of Tryptophan Metabolism in Health and Disease. Cell Host Microbe (2018) 23(6):716–24. 10.1016/j.chom.2018.05.003 29902437

[148] Yokoyama T Ph D Carlson R. Microbial Metabolites of Tryptophan Intestinal Tract With Special Reference. Am J Clin Nutr (1979) 32(1):173–8. 10.1093/ajcn/32.1.173 367144

[149] Whitehead TR Price NP Drake HL Cotta MA. Catabolic Pathway for the Production of Skatole and Indoleacetic Acid by the Acetogen Clostridium Drakei, Clostridium Scatologenes, and Swine Manure. Appl Environ Microbiol (2008) 74(6):1950–3. 10.1128/AEM.02458-07 PMC2268313 18223109

[150] Smith EA Macfarlane GT. Enumeration of Human Colonie Bacteria Producing Phenolic and Indolic Compounds: Effects of pH, Carbohydrate Availability and Retention Time on Dissimilatory Aromatic Amino Acid Metabolism. J Appl Bacteriol (1996) 81(3):288–302. 10.1111/j.1365-2672.1996.tb04331.x 8810056

[151] Gao J Xu K Liu H Liu G Bai M Peng C. Impact of the Gut Microbiotaon Intestinal Immunity Mediated by Tryptophan Metabolism. Front CellInfect Microbiol (2018) 8(FEB):1–22. 10.3389/fcimb.2018.00013 PMC5808205 29468141

[152] Elsden SR Hilton MG Waller JM. The End Products of the Metabolism of Aromatic Amino Acids by Clostridia. Arch Microbiol (1976) 107(3):283–8. 10.1007/BF00425340 1275638

[153] Lee JH Wood TK Lee J. Roles of Indole as an Interspecies and Interkingdom Signaling Molecule. Trends Microbiol (2015) 23(11):707–18. 10.1016/j.tim.2015.08.001 26439294

[154] Roager HM Licht TR. Microbial Tryptophan Catabolites in Health and Disease. Nat Commun (2018) 9(1):1–10. 10.1038/s41467-018-05470-4 30120222 PMC6098093

[155] Russell WR Duncan SH Scobbie L Duncan G Cantlay L Calder AG. Major Phenylpropanoid-Derived Metabolites in the Human Gut can Arise From Microbial Fermentation of Protein. Mol Nutr Food Res (2013) 57(3):523–35. 10.1002/mnfr.201200594 23349065

[156] Wlodarska M Luo C Kolde R d’Hennezel E Annand JW Heim CE. Indoleacrylic Acid Produced by Commensal Peptostreptococcus Species Suppresses Inflammation. Cell Host Microbe (2017) 22(1):25–37.e6. 10.1016/j.chom.2017.06.007 28704649 PMC5672633

[157] Debnar-Daumler C Seubert A Schmitt G Heider J. Simultaneous Involvement of a Tungsten-Containing Aldehyde: Ferredoxin Oxidoreductase and a Phenylacetaldehyde Dehydrogenase in Anaerobic Phenylalanine Metabolism. J Bacteriol (2014) 196(2):483–92. 10.1128/JB.00980-13 PMC3911259 24214948

[158] Cervantes-Barragan L Chai JN Tianero MD Di Luccia B Ahern PP Merriman J. Lactobaccillus Reuteri Induces Gut Intraepitelial CD4+ Cd8aa+ T Cells. Science (2017) 357(6353):806–10. 10.1126/science.aah5825 PMC5687812 28775213

[159] Wilck N Matus MG Kearney SM Olesen SW Forslund K Bartolomaeus H. Salt-Responsive Gut Commensal Modulates TH17 Axis and Disease. Nature (2017) 551(7682):585–9. 10.1038/nature24628 PMC6070150 29143823

[160] Zelante T Iannitti RG Cunha C DeLuca A Giovannini G Pieraccini G. Tryptophan Catabolites From Microbiota Engage Aryl Hydrocarbon Receptor and Balance Mucosal Reactivity Via Interleukin-22. Immunity (2013) 39(2):372–85. 10.1016/j.immuni.2013.08.003 23973224

[161] Aragozzini F Ferrari A Pacini N Gualandris R. Indole-3-lactic Acid as a Tryptophan Metabolite Produced by Bifidobacterium Spp. Appl Environ Microbiol (1979) 38(3):544–6. 10.1128/AEM.38.3.544-546.1979 PMC243529 533277

[162] Liu WH Chen FF Wang CE Fu HH Fang XQ Ye JR. Indole-3-Acetic Acid in Burkholderia Pyrrocinia JK-SH007: Enzymatic Identification of the Indole-3-Acetamide Synthesis Pathway. Front Microbiol (2019) 10:2559. 10.3389/fmicb.2019.02559 31749788 PMC6848275

[163] Williams BB Van Benschoten AH Cimermancic P Donia MS Zimmermann M Taketani M. Discovery and Characterization of Gut Microbiota Decarboxylases That can Produce the NeurotransmitterTryptamine. Cell Host Microbe (2014) 16(4):495–503. 10.1016/j.chom.2014.09.001 PMC4260654 25263219

[164] Li G Young KD. Indole Production by the Tryptophanase TnaA in Escherichia Coli is Determined by the Amount of Exogenous Tryptophan. Microbiol (United Kingdom) (2013) 159(2):402–10. 10.1099/mic.0.064139-0 23397453

[165] Keszthelyi D Troost FJ Masclee AAM. Understanding the Role of Tryptophan and Serotonin Metabolism in Gastrointestinal Function. Neurogastroenterol Motil (2009) 21(12):1239–49. 10.1111/j.1365-2982.2009.01370.x 19650771

[166] Vyhlídalová B Krasulová K Pečinková P Marcalíková A Vrzal R Zemánková L. Gut Microbial Catabolites of Tryptophan are Ligands and Agonists of the Aryl Hydrocarbon Receptor: A Detailed Characterization. Int J Mol Sci (2020) 21(7):2614. 10.3390/ijms21072614 PMC7177849 32283770

[167] Stepankova M Bartonkova I Jiskrova E Vrzal R Mani S Kortagere S. Methylindoles and Methoxyindoles are Agonists and Antagonists of Human Aryl Hydrocarbon Receptor. Mol Pharmacol (2018) 93(6):631–44. 10.1124/mol.118.112151 PMC5941192 29626056

[168] Marsland BJ. Regulating Inflammation With Microbial Metabolites. Nat Med (2016) 22(6):581–3. 10.1038/nm.4117 27270775

[169] Hashimoto T Perlot T Rehman A Trichereau J Ishiguro H Paolino M. ACE2 Links Amino Acid Malnutrition to Microbial Ecology and Intestinal Inflammation. Nature (2012) 487(7408):477–81. 10.1038/nature11228 PMC7095315 22837003

[170] Gutiérrez-Vázquez C Quintana FJ. Regulation of the Immune Response by the Aryl Hydrocarbon Receptor. Immunity (2018) 48(1):19–33. 10.1016/j.immuni.2017.12.012 29343438 PMC5777317

[171] Qiu Z Cervantes JL Cicek BB Mukherjee S Venkatesh M Maher LA. Pregnane X Receptor Regulates Pathogen-Induced Inflammation and Host Defense Against an Intracellular Bacterial Infection Through Toll-Like Receptor 4. Sci Rep (2016) 6(July):1–11. 10.1038/srep31936 27550658 PMC4994038

[172] Hakkola J Rysä J Hukkanen J. Regulation of Hepatic Energy Metabolism by the Nuclear Receptor PXR. Biochim Biophys Acta - Gene Regul Mech (2016) 1859(9):1072–82. 10.1016/j.bbagrm.2016.03.012 27041449

[173] Bock KW. Human and Rodent Aryl Hydrocarbon Receptor (AHR): From Mediator of Dioxin Toxicity to Physiologic AHR Functions and Therapeutic Options. Biol Chem (2017) 398(4):455–64. 10.1515/hsz-2016-0303 27805907

[174] Venkatesh M Mukherjee S Wang H Li H Sun K Benechet AP. Symbiotic Bacterial Metabolites Regulate Gastrointestinal Barrier Function Via the Xenobiotic Sensor PXR and Toll-Like Receptor 4. Immun (2014) 41(2):296–310. 10.1016/j.immuni.2014.06.014 PMC4142105 25065623

[175] Jennis M Cavanaugh CR Leo GC Mabus JR Lenhard J Hornby PJ. Microbiota-Derived Tryptophan Indoles Increase After Gastric Bypass Surgery and Reduce Intestinal Permeability In Vitro and In Vivo. Neurogastroenterol Motil (2018) 30(2):1–12. 10.1111/nmo.13178 28782205

[176] Lamas B Richard ML Leducq V Pham HP Michel ML Da Costa G. CARD9 Impacts Colitis by Altering Gut Microbiota Metabolism of Tryptophan Into Aryl Hydrocarbon Receptor Ligands. Nat Med (2016) 22(6):598–605. 10.1038/nm.4102 27158904 PMC5087285

[177] Lamas B Richard ML Sokol H. Caspase Recruitment Domain 9, Microbiota, and Tryptophan Metabolism: Dangerous Liaisons in Inflammatory Bowel Diseases. Curr Opin Clin Nutr Metab Care (2017) 20(4):243–7. 10.1097/MCO.0000000000000382 28399013

[178] Nikolaus S Schulte B Al-Massad N Thieme F Schulte DM Bethge J. Increased Tryptophan Metabolism Is Associated With Activity of Inflammatory Bowel Diseases. Gastroenterology (2017) 153(6):1504–1516.e2. 10.1053/j.gastro.2017.08.028 28827067

[179] Dinallo V Marafini I Fusco D Grazia A Laudisi F Dwairi R. Protective Effects Pf Aryl Hydrocarbon Receptor Signaling in Celiac Diesase Mucosa and in Poly L: C-induced Small Intestinal Astropy Mouse Model. Front Immunol (2019) 10(FEB):1–9. 10.3389/fimmu.2019.00091 30778350 PMC6369162

[180] Lamas B Hernández-Galan L Galipeau H Constante M Clarizio A Jury J. Aryl Hydrocarbon Receptor Ligand Production by the Gut Microbiota is Decreased in Celiac Disease Leading to Intestinal Inflammation. Sci Transl Med (2020) 12(566):eaba0624. 10.1126/scitranslmed.aba0624 33087499

[181] Stockinger B Meglio P Gialitakis M Duarte JH. The Aryl Hydrocarbon Receptor: Multitasking in the Immune System. Annu Rev Immunol (2014) 32:403–32. 10.1146/annurev-immunol-032713-120245 24655296

[182] Dong F Hao F Murray IA Smith PB Koo I Tindall AM. Intestinal Microbiota-Derived Tryptophan Metabolites are Predictive of Ah Receptor Activity. Gut Microbes (2020) 12:1–24. 10.1080/19490976.2020.1788899 PMC7524359 32783770

[183] Pernomian L Duarte-Silva M de Barros Cardoso CR. The Aryl Hydrocarbon Receptor (AHR) as a Potential Target for the Control of Intestinal Inflammation: Insights From an Immune and Bacteria Sensor Receptor. Clin Rev Allergy Immunol (2020) 59(3):382–90. 10.1007/s12016-020-08789-3 32279195

[184] Quintana FJ Sherr DH. Aryl Hydrocarbon Receptor Control of Adaptive Immunity. Pharmacol Rev (2013) 65(4):1148–61. 10.1124/pr.113.007823 PMC3799235 23908379

[185] ]Veldhoen M Ferreira C. Influence of Nutrient-Derived Metabolites on Lymphocyte Immunity. Nat Med (2015) 21(7):709–18. 10.1038/nm.3894 26121194

[186] Qiu J Zhou L. Aryl Hydrocarbon Receptor Promotes Rorγt+ Group 3 ILCs and Controls Intestinal Immunity and Inflammation. Semin Immunopathol (2013) 35(6):657–70. 10.1007/s00281-013-0393-5 PMC3797199 23975386

[187] Qiu J Guo X Chen Z ming E He L Sonnenberg GF Artis D. Group 3 Innate Lymphoid Cells Inhibit T-cell-mediated Intestinal Inflammation Through Aryl Hydrocarbon Receptor Signaling and Regulation of Microflora. Immunity (2013) 39(2):386–99. 10.1016/j.immuni.2013.08.002 PMC3884586 23954130

[188] Marinelli L Martin-Gallausiaux C Bourhis JM Béguet-Crespel F Blottière HM Lapaque N. Identification of the Novel Role of Butyrate as AhR Ligand in Human Intestinal Epithelial Cells. Sci Rep (2019) 9(1):1–14. 10.1038/s41598-018-37019-2 30679727 PMC6345974

[189] Hubbard TD Murray IA Perdew GH. Indole and Tryptophan Metabolism: Endogenous and Dietary Routes to Ah Receptor Activation. Drug Metab Dispos (2015) 43(10):1522–35. 10.1124/dmd.115.064246 PMC4576673 26041783

[190] Murray IA Nichols RG Zhang L Patterson AD Perdew GH. Expression of the Aryl Hydrocarbon Receptor Contributes to the Establishment of Intestinal Microbial Community Structure in Mice. Sci Rep (2016) 6(May):1–14. 10.1038/srep33969 27659481 PMC5034278

[191] Cervantes-Barragan L Colonna M. AHR Signaling in the Development and Function of Intestinal Immune Cells and Beyond. Semin Immunopathol (2018) 40(4):371–7. 10.1007/s00281-018-0694-9 29951906

[192] Zhu J Luo L Tian L Yin S Ma X Cheng S. Aryl Hydrocarbon Receptor Promotes IL-10 Expression in Inflammatory Macrophages Through Src-STAT3 Signaling Pathway. Front Immunol (2018) 9(SEP):1–14. 10.3389/fimmu.2018.02033 30283437 PMC6156150

[193] Chen W Pu A Sheng B Zhang Z Li L Liu Z. Aryl Hydrocarbon Receptor Activation Modulates CD8αα+Tcrαβ+ IELs and Suppression of Colitis Manifestations in Mice. BioMed Pharmacother (2017) 87:127–34. 10.1016/j.biopha.2016.12.061 28049094

[194] Brawner KM Yeramilli VA Duck LW Van Der Pol W Smythies LE Morrow CD. Depletion of Dietary Aryl Hydrocarbon Receptor Ligands Alters Microbiota Composition and Function. Sci Rep (2019) 9(1):1–12. 10.1038/s41598-019-51194-w 31604984 PMC6789125

[195] Scott SA Fu J Chang PV. Microbial Tryptophan Metabolites Regulate Gut Barrier Function Via the Aryl Hydrocarbon Receptor. Proc Natl Acad Sci U S A (2020) 117(32):19376–87. 10.1073/pnas.2000047117 PMC7431026 32719140

[196] Fukumoto S Toshimitsu T Matsuoka S Maruyama A Oh-Oka K Takamura T. Identification of a Probiotic Bacteria-Derived Activator of the Aryl Hydrocarbon Receptor That Inhibits Colitis. Immunol Cell Biol (2014) 92(5):460–5. 10.1038/icb.2014.2 24518984

[197] Yin J Yang K Zhou C Xu P Xiao W Yang H. Aryl Hydrocarbon Receptor Activation Alleviates Dextran Sodium Sulfate-Induced Colitis Through Enhancing the Differentiation of Goblet Cells. Biochem Biophys Res Commun (2019) 514(1):180–6. 10.1016/j.bbrc.2019.04.136 31029423

[198] Liu Z Li L Chen W Wang Q Xiao W Ma Y. Aryl Hydrocarbon Receptor Activation Maintained the Intestinal Epithelial Barrier Function Through Notch1 Dependent Signaling Pathway. Int J Mol Med (2018) 41(3):1560–72. 10.3892/ijmm.2017.3341 PMC5819918 29286081

[199] Yu K Ma Y Zhang Z Fan X Li T Li L. Ahr Activation Protects Intestinal Epithelial Barrier Function Through Regulation of Par-6. J Mol Histol (2018) 49(5):449–58. 10.1007/s10735-018-9784-1 29992488

[200] Yu M Wang Q Ma Y Li L Yu K Zhang Z. Aryl Hydrocarbon Receptor Activation Modulates Intestinal Epithelial Barrier Function by Maintaining Tight Junction Integrity. Int J Biol Sci (2018) 14(1):69–77. 10.7150/ijbs.22259 29483826 PMC5821050

[201] Fang L Pang Z Shu W Wu W Sun M Cong Y. Anti-TNF Therapy Induces CD4 + T-Cell Production of IL-22 and Promotes Epithelial Repairs in Patients With Crohn’s Disease. Inflammation Bowel Dis (2018) 24(9):1733–44. 10.1093/ibd/izy126 29718341

[202] Zhao Y Ma T Chen W Chen Y Li M Ren L. MicroRNA-124 Promotes the Intestinal Inflammation by Targeting AHR in Crohn’s Disease. (2016) J Crohns Colitis 10(6):703–12. 10.1093/ecco-jcc/jjw010 26802080

[203] Chng SH Kundu P Dominguez-Brauer C Teo WL Kawajiri K Fujii-Kuriyama Y. Ablating the Aryl Hydrocarbon Receptor (AhR) in CD11c+ Cells Perturbs Intestinal Epithelium Development and Intestinal Immunity. Sci Rep (2016) 6(October 2015):2–3. 10.1038/srep23820 27068235 PMC4828637

[204] Riemschneider S Hoffmann M Slanina U Weber K Hauschildt S Lehmann J. Indol-3-carbinol and Quercetin Ameliorate Chronic Dss-Induced Colitis in c57bl/6 Mice by Ahr-Mediated Anti-Inflammatory Mechanisms. Int J Environ Res Public Health (2021) 18(5):1–17. 10.3390/ijerph18052262 PMC7956562 33668818

[205] Biagioli M Carino A Fiorucci C Annunziato G Marchiano S Bordoni M. The Aryl Hydrocarbon Receptor (Ahr) Mediates the Counter-Regulatory Effects of Pelargonidins in Models of Inflammation and Metabolic Dysfunctions. Nutrients (2019) 11(8):1820. 10.3390/nu11081820 PMC6723439 31394746

[206] Kimura A Naka T Nakahama T Chinen I Masuda K Nohara K. Aryl Hydrocarbon Receptor in Combination With Stat1 Regulates LPS-induced Inflammatory Responses. J Exp Med (2009) 206(9):2027–35. 10.1084/jem.20090560 PMC2737163 19703987

[207] Na YR Stakenborg M Seok SH Matteoli G. Macrophages in Intestinal Inflammation and Resolution: A Potential Therapeutic Target in IBD. Nat Rev Gastroenterol Hepatol (2019) 16(9):531–43. 10.1038/s41575-019-0172-4 31312042

